# Rare‐Earth‐Metal‐Free Solid‐State Fluorescent Carbonized‐Polymer Microspheres for Unclonable Anti‐Counterfeit Whispering‐Gallery Emissions from Red to Near‐Infrared Wavelengths

**DOI:** 10.1002/advs.202400693

**Published:** 2024-06-12

**Authors:** Barun Kumar Barman, Hiroyuki Yamada, Keisuke Watanabe, Kenzo Deguchi, Shinobu Ohki, Kenjiro Hashi, Atsushi Goto, Tadaaki Nagao

**Affiliations:** ^1^ Research Center for Materials Nanoarchitectonics (WPI‐MANA) National Institute for Materials Science (NIMS) Tsukuba Ibaraki 305‐0044 Japan; ^2^ Research Network and Facility Services Division National Institute for Materials Science (NIMS) 3‐13 Sakura Tsukuba Ibaraki 305‐0003 Japan; ^3^ Center for Basic Research on Materials National Institute for Materials Science (NIMS) 3‐13 Sakura Tsukuba Ibaraki 305‐0003 Japan; ^4^ Department of Condensed Matter Physics Graduate School of Science Hokkaido University Sapporo Hokkaido 060‐0810 Japan

**Keywords:** carbonized polymer microsphere, encoded‐anti‐counterfeit, near‐infrared, solid‐state emission, whispering‐gallery mode

## Abstract

Colloidal carbon dots (CDs) have garnered much attention as metal‐free photoluminescent nanomaterials, yet creation of solid‐state fluorescent (SSF) materials emitting in the deep red (DR) to near‐infrared (NIR) range poses a significant challenge with practical implications. To address this challenge and to engineer photonic functionalities, a micro‐resonator architecture is developed using carbonized polymer microspheres (CPMs), evolved from conventional colloidal nanodots. Gram‐scale production of CPMs utilizes controlled microscopic phase separation facilitated by natural peptide cross‐linking during hydrothermal processing. The resulting microstructure effectively suppresses aggregation‐induced quenching (AIQ), enabling strong solid‐state light emission. Both experimental and theoretical analysis support a role for extended π‐conjugated polycyclic aromatic hydrocarbons (PAHs) trapped within these microstructures, which exhibit a progressive red shift in light absorption/emission toward the NIR range. Moreover, the highly spherical shape of CPMs endows them with innate photonic functionalities in combination with their intrinsic CD‐based attributes. Harnessing their excitation wavelength‐dependent photoluminescent (PL) property, a single CPM exhibits whispering‐gallery modes (WGMs) that are emission‐tunable from the DR to the NIR. This type of newly developed microresonator can serve as, for example, unclonable anti‐counterfeiting labels. This innovative cross‐cutting approach, combining photonics and chemistry, offers robust, bottom‐up, built‐in photonic functionality with diverse NIR applications.

## Graphical abstract

Bio‐inspired carbonized polymer microresonators is developed to generate whispering‐gallery emission in the deep red to near‐infrared wavelength range, effectively suppressing aggregation‐induced quenching. The extended π‐conjugated polycyclic aromatic hydrocarbons trapped within these microstructures lead to a progressive red shift in light absorption/emission toward the NIR range. These microresonators enable versatile applications such as unclonable, anti‐counterfeiting ink with integrated photonic functionalities.

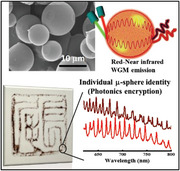

## 1 Introduction

Carbon dots (CDs) and carbonized polymer dots (CPDs) have emerged as luminescent nanoparticles with singular optical properties, eco‐friendly synthesis, and low toxicity.^[^ ^1^ ^]^ They have attracted great attention in lighting applications due to their excellent biocompatibility, stable emission, and potential to replace costly, toxic semiconductor quantum dots (CdS, CdSe, CdTe, PbS, PbSe etc.), as well as rare earth‐doped oxynitride (SiAlON)‐based ceramic phosphors. Despite improvements in high photoluminescence quantum yields (PLQY) in colloidal solution, their practical application encounters challenges such as aggregation‐induced quenching (AIQ), attributed to π–π interactions and resonance energy transfer, often observed in solid‐state phosphors.^[^ ^2^ ^]^ Strategies involving integration of CDs into matrices like polymers or salts seek to overcome these hurdles, yet issues including inadequate photostability and uneven dispersion persist.^[^ ^3^ ^]^ Ongoing efforts focus on developing CDs with AIQ‐resistant solid‐state fluorescence by employing structural design approaches to mitigate π–π stacking and excessive Förster resonance energy transfer (FRET) without using external substances.^[^ ^4^ ^]^ Moreover, developing CDs capable of efficient long‐wavelength emissions, especially in the deep red to nearinfrared (DR‐NIR) spectral regions (>700 nm), poses a significant challenge, despite their vast potential for applications in security, biodetection, bio‐imaging, and photodynamic/photothermal therapy.^[^ ^5^ ^]^ In wearable or implanted biosensors reliant on NIR photons for vital sign monitoring and wireless communication, addressing the challenge of efficient long‐wavelength emissive CDs is pivotal.^[^ ^6^ ^]^

Overcoming hurdles in generating efficient DR‐NIR emission involve reducing energy gaps and reducing non‐radiative decay of CDs.^[^ ^6^, ^7^ ^]^ While carbon nanotubes (CNTs) exhibit high emission potential, their chirality dependence complicates controlled optimization. Biocompatibility concerns, complex surface functionalization, manufacturing complexities, and regulatory uncertainties impede CNT utilization.^[^ ^8^ ^]^ Despite these challenges, CDs surpass inorganic counterparts due to their flexibility, conformability, and cost‐effectiveness. Ongoing research shows that CDs offer promising solutions for creating solid‐state, NIR emitters that are compatible with biological systems as well as highly sustainable synthesis methods. This advancement is particularly significant for applications in biomedicine and the food industry, especially for uses involving detection and imaging in the NIR.^[^ ^9^ ^]^ While CDs have been utilized in various applications, including as laser gain media through integrated microcavities,^[^ ^10^ ^]^ their inbuilt photonic functionality in the VIS‐NIR region remains unexplored. Therefore, investigating the interaction of light with CD‐based microstructures could advance lighting technology, bioimaging, and nano‐medical applications in the near future.

The photonic behavior of nano to microstructures heavily depends on their shape and size. Typical PL spectra of both organic and inorganic semiconductor quantum dots (QDs), as well as phosphor particles, typically exhibit a Gaussian fluorescence pattern when excited electronically. However, if these particles possess curved or reflective surfaces that act as mirrors, they can also function as photonic resonators by enabling multiple total internal reflections (TIR) of light. Due to TIR, interference of light waves may lead to generation of optical, whispering‐gallery‐mode (WGM) resonances within the microstructure.^[^ ^11^ ^]^These resonances appear as a series of sharply intensified lines (modes) in the PL spectrum. While self‐assembled, organic microparticles consisting of dyes, π‐conjugated polymers,^[^ ^12^ ^]^ and superstructures of inorganic QDs have shown the WGM effect,^[^ ^13^ ^]^ there has been no scientific exploration of the photonic properties of materials based on carbon dots (CDs). Hence, this study sought to discover intrinsic solid‐state “photonic” characteristics of CD‐based microstructures, with the objective of engineering red to NIR emissions.

**Figure 1:**
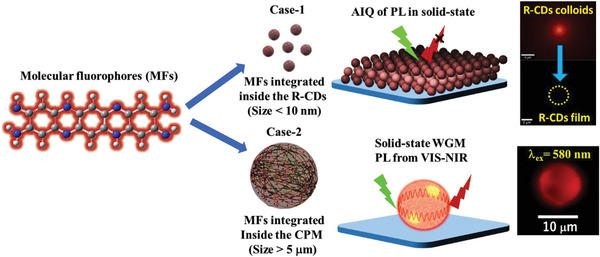
Integration of molecular fluorophores (MFs) into red‐emitting carbon dots (R‐CDs) and red‐emitting carbonized polymer microspheres across nano‐ to micrometer scales. In Case‐1, AIQ of PL from colloidal and solid‐state R‐CDs. In Case‐2, MFs are confined within microspheres, resulting in solid‐state WGM emission spanning VIS to NIR wavelengths, as evidenced by an optical micrograph.

Our innovation diverges from traditional nanodot synthesis, constructing carbonized polymer microspheres (CPM) by chemically fine‐tuning π‐conjugated polycyclic aromatic hydrocarbons (PAHs) inside microspheres (**Figure** **1**). CPM synthesis on a gram scale involves natural peptide cross‐linking during hydrothermal processing, inducing microscopic phase separation. Creating these microstructures effectively blocks AIQ, paving the way for solid‐state light emission. Both experimental observations and theoretical frameworks demonstrate a noticeable red shift in light absorption, along with elongated emission in the NIR range due to the extended π‐conjugation of PAHs confined within these microstructures. With a distinctive spherical form, CPMs exhibit inherent photonic properties as well as characteristics based on intrinsic CDs. Their PL properties, dependent on excitation wavelength, enable tunable microresonator emissions from the visible to the NIR range (up to 1000 nm), making CPMs more versatile than traditional semiconductor and dye‐based microresonators.^[^ ^12^, ^14^ ^]^ A single CPM displays adjustable, sharp WGM emission across a broad spectrum by altering the excitation source. These microresonators can be swiftly applied as secure anti‐counterfeiting labels in diverse fields due to their specific excitation wavelength‐dependent microresonator emissions. Fusion of CPM‐generated, red‐NIR emissions with microcavity‐associated WGM light emissions unveils a multitude of applications spanning bioimaging, sensing, and photonics applications.

## 2 Results and Discussion

CDs/CPDs can be considered as blends of different fluorescent centers arising from various molecular fluorophores (MFs) or their aggregates. However, their PL diminishes in the solid‐state due to AIQ, as evidenced in optical micrographs showing the transition from colloidal to solid‐state for red‐emitting carbon dots (R‐CDs) under 532 nm laser excitation (Case I in Figure 1). Contrastingly, in the CPM configuration, MFs are confined and isolated inside microspheres, leading to solid‐state WGM emissions covering VIS to NIR wavelengths. This is evident when excited by a 580 nm laser, as shown in an optical micrograph (Case II in Figure 1).

**Figure 2:**
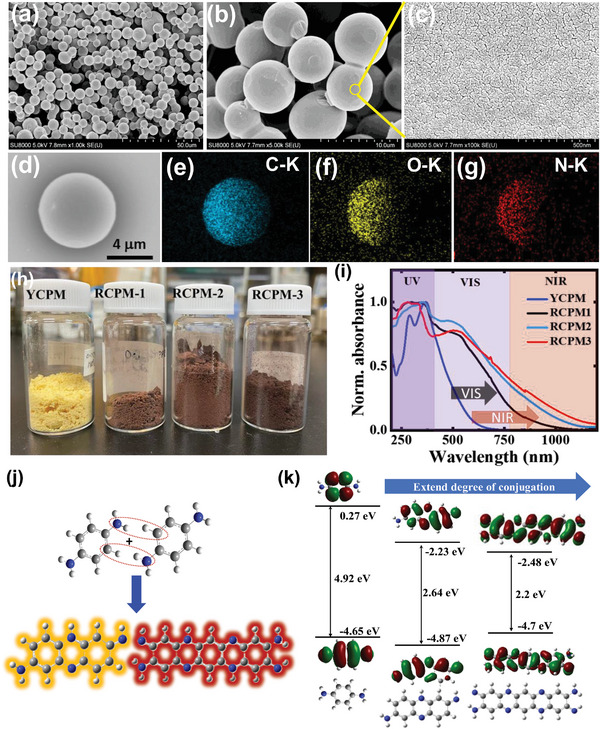
a–c) SEM images of microspheres and surface texture of RCPM‐2. EDS mapping in a single RCPM‐2 is depicted in (d–g). h) A photograph demonstrating controlled conversion from YCPM to RCPM through PAH regulation. i) UV–vis–NIR absorbance spectra of YCPM to RCPM1‐3. j) DFT calculation results for PAH models with increasing conjugation, including computed energy diagrams for the HOMO‐LUMO energy gap.

R‐CDs were synthesized through a solvothermal reaction involving p‐phenylenediamine (PPD) in N,N‐diethylformamide solvent. Meanwhile, DR‐NIR red‐emitting carbonized polymeric microspheres (RCPMs) were produced using a hydrothermal method that utilizes PPD and citric acid (CA), with ε‐polylysine (EPL) linear peptides. This approach mimics the denaturation process observed in natural proteins or peptides (Figure S1, Supporting Information). Carbon microspheres (CMs) offer a comparison, synthesized in a hydrothermal reaction via carbonizing CA and glucose, respectively (details in the experimental section). Scanning electron microscopy (SEM) and energy dispersive spectroscopy (EDS) are used to assess the morphology and elemental composition of RCPMs. SEM images depict RCPM‐2 microspheres exhibiting a consistent diameter (5–15 µm) (**Figure** **2**a,b). Further examination at higher magnification reveals nonporous textures, displaying assemblies of nano‐sized CDs (Figure 2c). SEM images of YCPM, RCPM‐1, and RCPM‐3 (Figure S2, Supporting Information), and carbon spheres (CM) show microsphere formation (Figure S3, Supporting Information). The surface texture of RCPM‐3 becomes rougher and deformed compared to RCPM1‐2. EDS elemental mapping of an individual microsphere confirms the primary composition of RCPM‐2 as Carbon (C), Oxygen (O), and Nitrogen (N) (Figure 2d,e). EPL, cross‐linked by CA, forms yellow‐colored CPMs (YCPMs) with gram‐scale microsphere formation. Increasing PPD content 6–22 wt.% transforms yellow (YCPMs) to dark reddish‐brown RCPMs (Figure 2h). The transition from yellow to dark brown signifies transformation of material properties in the VIS‐NIR absorption. The YCPM absorbance spectrum shows peaks at 280 nm and ≈370 nm, with broad visible absorbance. The strong UV absorbance is attributed to π–π^*^ and n–π^*^ transitions of C─O and C─N bonding configurations from trapped blue‐light‐emitting MF derivatives such as 5‐oxo‐1,2,3,5‐tetrahydro‐imidazo[1,2‐α] pyridine‐7‐carboxylic acid (IPCA),^[^ ^15^ ^]^ possibly formed via the reaction between CA and EPL (Figure 2i). Additionally, the lower energy absorbance band is due to n–π^*^ transitions of cross‐linked amide bond structures involving C═O and C═N bonds.^[^ ^16^ ^]^ RCPMs exhibit red‐shifted absorbance from UV to vis–NIR and with a gradual increase of PPD, further extending the absorbance spectrum from the visible to the NIR range (Figure 2i). Currently, researchers have discovered that incorporation of PPD molecules leads to formation of red emitting CDs^[^ ^17^ ^]^ This emission is assigned to formation of pyrazine‐like, red‐light‐emitting PAH‐based MFs in resulting R‐CDs (Figure 2j).^[^ ^17^, ^18^ ^]^ To understand the optical mechanism of RCPMs, Density Functional Theory (DFT) calculations are currently being performed on three distinct PAH structures. Geometric optimization and energy level calculations are conducted, revealing HOMO‐LUMO gaps of 2.64 and 2.2 eV of the PAHs, respectively (Figure 2k). Simulated absorbance spectra of MFs of PAHs also exhibit red shifts in absorbance, attributed to narrowing of the optical gap caused by the increased extent of π conjugation (Figure S4, Supporting Information). The gradual increase in absorbance from the UV to NIR spectral range corresponds to the gradual entrapment and enhancement of PAHs, transitioning from YCPMs to RCPMs1‐3, as the wt.% of PPD increases during synthesis. The reaction involving CA and EPL to trap IPCA MFs results in bandgaps corresponding to the UV‐region (YCPM), whereas integration of conjugated PAH segments shifts their bandgaps from visible wavelengths to the NIR region (Figure 2i).

**Figure 3:**
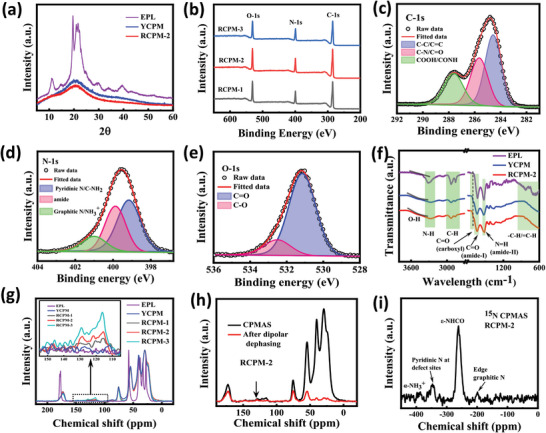
a) XRD patterns of EPL, YCPMs, and RCPMs‐2. b) XPS survey spectra of different RCPMs1‐3. c–e) Deconvoluted HRXPS spectra of C‐1s, N‐1s, and O‐1s of RCPMs‐2. f) FTIR spectra of EPL, YCPMs, and RCPMs‐2. g) Solid‐state ^13^C CPMAS NMR spectra of different samples and compared with the EPL (The inset shows magnified NMR spectra). h) ^13^C CPMAS NMR (black) and dipolar de‐phased multi‐CP spectra (red line) of protonated and non‐protonated carbon spectra of RCPMs‐2. i) ^15^N CPMAS experimental spectra of RCPMs‐2.

X‐ray diffraction (XRD) patterns show a broad peak corresponding to CPM (YCPM and RCPM2) structures at a 2*θ* value of 21°, along with the absence of a sharp peak associated with the ordered peptide (EPL) (**Figure** **3**a).^[^ ^19^ ^]^ This absence indicates formation of an amorphous structure, most likely due to cross‐linking polymeric structures in the material.^[^ ^16a^ ^]^ X‐ray photoelectron spectroscopy (XPS) survey spectra reveal the presence of C, O, and N in RCPMs1‐3 (Figure 3b). The atomic ratio observed in YCPMs and RCPMs1‐3 obtained from XPS survey spectra shows an increase in O content and a decrease in N content compared to EPL (Figure S5, Supporting Information). This is attributed to formation of amide bonds resulting from the reaction between CA and EPL (Table S2, Supporting Information). The N/C ratio of RCPMs gradually increases compared to YCPMs (Table S2, Supporting Information) due to additional PAHs incorporated into the RCPMs. Deconvoluted, high‐resolution XPS (HRXPS) spectra of C 1s display different types of C chemical bonds (C─C/C═C, C─N/C═O, COOH/CONH), indicating that various functional groups are present in RCPMs‐2 (Figure 3c). The deconvoluted high‐resolution XPS (HRXPS) spectrum of RCPMs‐2 for C‐1s exibit different types of C chemical bonding, indicating that various functional groups are present in the RCPMs2. The deconvoluted C‐1s peak consists of three types of C bonds: C─C/C═C, C─N/C─O, and COOH/CONH (Figure 3c). N‐1s HRXPS results exhibit a peak at 399.0 eV corresponding to C─NH_2_ and C─N─C (pyridinic nitrogen) and a peak at 400 eV due to the amide bond, and peak at 401.2 eV (graphitic) indicating graphitic N that demonstrates formation of a pyridinic bond, graphitic N, and large amide bonds (Figure 3d).^[^ ^20^ ^]^ In the O 1s region, two peaks at 531.2 and 532.6 eV attributed to the N─C═O and O─C═O bonds, respectively, are consistent with different O functionalities of O 1s in RCPMs‐2 (Figure 3e). De‐convolution of the C‐1s, N‐1s, and O‐1s spectra of YCPMs, RCPMs‐1, 3, and R‐CDs also reveal different functional groups (Figure S6, Supporting Information). Attenuated total reflection‐Fourier transform, infrared spectroscopy (ATR‐FTIR) spectra reveal that most functional groups observed are similar to those of EPL, such as peaks appearing at 3200 cm^−1^ (NH_2_) and 1630 cm^−1^ (amide C═O). But new broad feature or shoulder emerges with new brord bands at 3500 cm^−1^ (carboxyl O─H) and 1700 cm^−1^ (carboxyl C═O), which indicates that the EPL linear chain structure is reacted with CA containing ─COOH groups, leading to the formation of condensed spherical microstructure via the reaction of these functional groups (Figure 3f). The small sharp peak at 825 cm^−1^ can be assigned to the Ar─H bending modes corresponding to the PAHs (Figure S7, Supporting Information).^[^ ^17^ ^]^ RCPM structure was characterized by solid‐state nuclear magnetic resonance (ssNMR) using ^13^C and ^15^N. ^13^C cross‐polarization, magic angle spinning (CPMAS) NMR spectra of pure EPL, YCPMs, RCPMs1‐3 are shown in Figure 3g. Solid‐state ^13^C CPMAS NMR spectra of YCPM to RCPM‐1, 3 samples reveal distinct peaks, notably at ≈29 and 40 ppm, corresponding to sp^3^‐hybridized CH_2_ sequences in aliphatic C compared with the EPL. A broad peak at 173 ppm suggests a cross‐linked structure involving ─COOH groups from CA and ─NH_2_ groups from EPL, contributing to YCPM structures.^[^ ^21^ ^]^ Broadening of the aliphatic carbon (CH_2_) spectral pattern supports cross‐linking, and upfield shifts of the amorphous component indicate conformational heterogeneity.^[^ ^22^ ^]^ Peaks spanning 130 to 115 ppm suggest sp^2^‐hybridized carbon (3–7% of total intensity).^[^ ^23^ ^]^ The inset of Figure 3g shows gradual enhancement of these peaks, indicating formation of more PAHs inside RCPM structures. Bonding between C and H is evident through dipolar dephasing, with significant reductions in signal intensity of nonprotonated carbon ≈120 ppm in the dipolar‐dephased spectrum (Figure 3h). Another consistent peak at 129 ppm becomes more resolved after dipolar dephasing, indicating a carbon atom not bonded to hydrogen, resembling characteristics of a CH‐substituted aromatic carbon. An alternative interpretation suggests a substituted alkene; however, conjugated double bonds would be necessary.^[^ ^23^, ^24^ ^]^ Magnified ^13^C CP‐MAS NMR spectra also exhibit small peaks ≈148 ppm, likely originating from sp^2^‐hybridized carbon. The lower chemical shift at 85.5 ppm is indicative of ═CH carbons, influenced by the indirect electron‐withdrawing impact of two N atoms at a two‐bond distance (Figure S8, Supporting Information).^[^ ^23^ ^]^ The sp^2^‐hybridized carbon linked to two N atoms, along with the associated ═CH, suggests possible formation of IPCA derivatives confined to YCPM architectures. This result suggests formation of IPCA MFs being confined within microstructures via the reaction between CA and EPL (Figure S9, Supporting Information). In the ^15^N NMR spectrum (Figure 2i), peaks at 198, 261, 346, and 386 ppm correspond to edge graphitic N, main chain ε‐NHCO, α‐NH_3_^+^ group, and pyridinic N at defect sites, respectively.^[^ ^25^ ^]^ Combining these findings with ssNMR studies, we propose a plausible process to form RCPM structures, transitioning to NIR‐emissive states (Figure 1).

**Figure 4:**
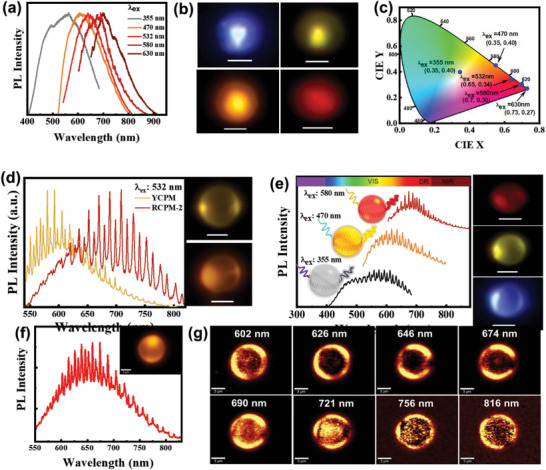
a,b) Excitation wavelength‐dependent PL spectrum and µ‐PL images by different excitation 355, 470, 532, and 580 nm laser. c) CIE coordinates corresponding to the emitted light. d) Comparison of WGM emission and corresponding µ‐PL images of YCPMs and RCPMs‐2 via 532‐nm laser excitation at their edges (scale bar 5 µm). e) vis–NIR range WGM emission and corresponding µ‐PL from an individual RCPM‐2 microresonator by varying the laser excitation at their edges. f,g) μ‐PL spectra and mapping images of a single microresonator and corresponding light confinement from DR‐NIR wavelengths.

The PL properties of RCPMs are noteworthy, exhibiting variation based on excitation wavelength across the vis–NIR spectrum. Upon excitation with different wavelengths (355, 470, 532, 580, and 630 nm), RCPMs‐2 display a fascinating light emission ranging from white to deep red. This transition is visually confirmed through micro‐photoluminescence (µ‐PL) images obtained with individual microspheres under different excitations (**Figure** **4**a,b). Corresponding emission colors are revealed in the CIE coordinates, providing a comprehensive overview of microsphere responses to distinct excitations (Figure 4c). Alternatively, the PL of R‐CDs diminishes as they shift from a colloidal state to a solid form, mainly attributable to AIQ (Figure S10, Supporting Information). While the absolute PL quantum yield (PLQY) of RCPMs‐1 remains ≈1.7% in the solid state, it contrasts with the PLQY of ≈6.8% observed in the R‐CDs colloidal solution by 530 nm excitation.

The 3‐D PL spectrum reveals that YCPMs show prominent emissions in the blue and green regions, with weaker yellow emission (Figure S11, Supporting Information). YCPMs exhibit multi‐color emissions spanning from cyan to yellow wavelengths, due to both MFs and cross‐linked enhanced emissive (CEE) centers embedded in the YCPM matrix. Upon excitation at 360 nm, a simultaneous double‐peak fluorescence emission at 446 and 508 nm is observed, resulting in cyan‐colored emissions. The 446 nm peak is associated with the presence of IPCA MF derivatives. The strong green emission and emissions at higher wavelengths can be ascribed to the CEE effect induced by amide bond formation.^[^ ^16^, ^26^ ^]^ This effect amplifies the luminescence of nonconjugated polymer by restricting bond vibrations and rotations upon immobilization of small molecular rotors, such as ─C═N, ─C═O, and other hetero‐molecules containing double bonds.^[^ ^26^, ^27^ ^]^ This constraint of bond movement within the polymer matrix enhances light emission by improving electron concentrations, thereby reducing energy loss through non‐radiative pathways. In the case of RCPMs, additional red‐emitting PAHs trapped in microstructures lead to a further red shift in emission, extending from visible to NIR wavelengths, compared to YCPMs. The μ‐PL spectrum under UV laser excitation (355 nm) demonstrated a significant decrease in emissions ≈450 nm and a simultaneous increase in emissions at ≈570–580 nm from YCPMs to RCPMs1‐3 (Figure S12a, Supporting Information). As a result, CIE coordinates and µ‐PL images of a single microsphere indicate broad emissions across the entire visible spectrum, resulting in white light emission (WLE) from RCPMs compared to YCPMs, which emit cyan light under UV excitation (Figure S12b–f, Supporting Information). By investigating time‐resolved photoluminescence (TRPL) at 449 nm excitation to probe FRET between MFs in CPMs, notable changes were observed. YCPMs exhibited a lifetime (*τ*_av_) of 7.345 ns (Figure S13a,b and Table S3, Supporting Information). Conversely, for the transition from YCPMs to red‐emitting chromophores (RCPMs‐1,2), *τ*_av_ decreased to 0.916 and 0.846 ns (Figure 13c,f and Table S3, Supporting Information), respectively. Observed differences in lifetimes indicated that energy transfer efficiencies from YCPMs to RCPMs‐1,2 were ≈88%. This compellingly suggests that incorporation of PAHs in RCPMs resulted in a dramatic enhancement of effective excitation energy transfer from blue light‐emitting MFs in YCPMs to pyrazine‐like PAHs. Moreover, the concentration of PAHs in RCPMs is important in tuning the emitted white light from cool white to natural white, progressing from RCPMs‐1 to RCPMs‐3 (Figure S12c,f, Supporting Information). A distinctive advantage of RCPMs over conventional carbon dots lies in their ability to maintain emission properties in the solid state without succumbing to AIQ. This resilience arises because the PAHs are contained in RCPMs, which prevents non‐radiative recombination caused by energy or charge transfer between different luminescent centers. As a result, RCPMs can emit light directly in the solid‐state across the vis–NIR spectral range.

Intriguingly, with efficient light‐trapping, these spherical CPMs serve as built‐in photonic microresonators. Upon excitation with a focused laser beam on their edges, CPMs can generate WGMs from which emitted PL is confined and resonates within the sphere^.[^ ^11^, ^12^, ^28^ ^]^ Remarkably, a single spherical RCPM demonstrates the ability to resonate across a broad range of wavelengths, spanning DR to NIR wavelengths, with emission wavelengths adjusted by simply varying the excitation wavelength. Figure 4d shows the μ‐PL spectra and corresponding μ‐PL image demonstrating emission of WGMs from the edge of the YCPM and RCPM‐2 microspheres. The distinct signature of an integrated microresonator is evident from PL spectra collected along the edge of an individual microsphere. This unique characteristic arises from efficient confinement of emitted photons in the spherical architecture. WGM emission clearly shifted from yellow wavelengths to the DR to NIR wavelengths from YCPMs to RCPMs, due to formation of red‐light emitting PAHs. When excited at wavelengths of 355, 470, and 580 nm, RCPMs‐2 emit WGMs spanning visible to NIR wavelengths (Figure 4e). In contrast, to YCPMs, WGM emissions are limited to cyan to red wavelengths, corresponding to μ‐PL emission (Figure S14a,b, Supporting Information). This indicates optical modulation of microresonators by modifying internal structures with hydrothermally formed PAHs. In comparison to RCPMs1 and RCPMs2, RCPMs‐3 exhibit a notable increase in surface roughness (Figure S2, Supporting Information) and absorbance (Figure 2i), thereby resulting in a significant reduction in light confinement through WGM resonance (Figure S15, Supporting Information). This reduction in light confinement can be attributed to internal losses arising from scattering at the rough surface and enhanced self‐absorption. The power dependence on WGM emissions in the DR‐NIR spectral range is also demonstrated, showing light confinement near the edges due to WGM resonance (Figure S16, Supporting Information). Localization of light at sphere edges is further confirmed through µ‐PL mapping of a single microresonator. Both the µ‐PL spectrum and µ‐PL mapping at various resonating wavelengths provide clear evidence of light confinement at micro‐resonator perimeters due to WGM formation (Figure 4f,g).

**Figure 5:**
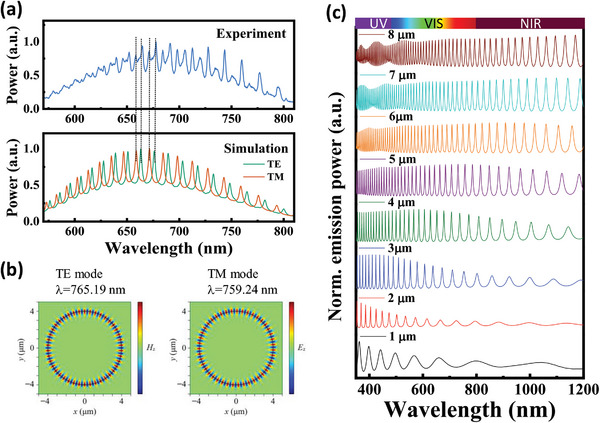
a,b) Experimental (top) and simulated (bottom) PL spectra excited at 532 nm, and electromagnetic field distributions of TE and TM resonances in the NIR region c) Simulated emissions in TM modes for various microsphere diameters.

Experimental PL spectra and field profiles were simulated using the finite‐difference time‐domain (FDTD) method. Simulated WGM resonances, combined with background emissions from RCPMs‐2, closely matched the experimental spectra (**Figure** **5**a) for a microresonator diameter (D) of 8 µm. Here, the spectra suggest contributions from both the transverse electric (TE) and transverse magnetic (TM) modes of the sphere (Figure 5a). The varying optical paths naturally lead to differences in mode separation for each type of WGM, resulting in a non‐uniform spectral separation of resonant peaks. Representative mode profiles at WGM resonance peaks ≈760 nm are shown in Figure 5b as a Hz profile for TE mode and an Ez profile for the TM mode, respectively, representing characteristics of fundamental TE and TM modes with a radial mode number of 1. In Figure 5c, simulated TM mode profiles of the microresonator are shown for diameters ranging from 1 to 8 µm. The minimum diameter sphere detectable for visible wavelengths is expected to be ≈2 µm, considering that emission wavelengths are larger than wavelengths of the absorption peak (λ ≈370 nm). When the diameter is smaller than 2 µm, fundamental TE or TM modes cannot be tightly confined inside the sphere, leading to the disappearance of resonance modes due to a failure to satisfy the resonant condition *n*_eff_πD = mλ (where m represents the azimuthal order of the mode and *n*_eff_ is the effective refractive index). To observe WGM resonance at NIR wavelengths, microresonator diameters larger than 3 µm are required to confine the light. As these RCPM diameters are larger, i.e., D > 5 µm, both visible and NIR emitted light can be tightly confined and WGM emissions are observed experimentally.

**Figure 6:**
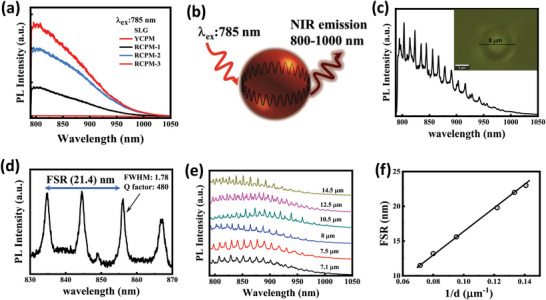
a) NIR emission profiles of RCPMs‐1, RCPMs‐2, YCPMs and a soda lime glass substrate (SLG) at 785 nm laser excitation. b) A schematic illustration of WGM NIR emissions generated through edge excitation of individual microspheres. c) NIR WGM emissions from a single RCPM‐2. The inset shows an optical image of the microsphere. d) Q factor and free spectral range (FSR) at NIR wavelengths from a high‐resolution μ‐PL spectrum. e,f) Diameter‐dependent WGM emissions and their correlation with the corresponding FSR.

In contrast to YCPMs, which lack DR‐NIR, RCPMs‐2 display a prominent NIR emission peak at 820 nm when excited with a 785 nm laser (**Figure** **6**a). This exceptional property results in PLQY of ≈2.2% at NIR wavelengths in the solid state. The comparative μ‐PL spectrum reveals that typical carbon spheres (CSs) derived from mixtures of sucrose and CA exhibit weaker NIR emission, whereas RCPMs‐2 demonstrate ≈20 times higher NIR emission (Figure S17, Supporting Information). When excited at 785 nm on their edges, NIR WGM emission is observed, extending up to 1000 nm (Figure 6b,c). Remarkably, the quality factor (Q = λ/Δλ, where λ is the resonant wavelength, and Δλ is the full width at half‐maximum (FWHM) of the resonance) of RCPMs‐2 can reach as high as 480 at a WGM wavelength of 856 nm in the NIR range calculated from high resolution PL spectra (Figure 6d). Figure 6e shows μ‐PL spectra of different diameter microresonators (Figure S18, Supporting Information). The free spectral range (FSR), which is the difference between two consecutive resonances is inversely correlated to the cavity diameter (FSR = $\lambda_{m}^{2}$/*n*_eff_πD).^[^ ^11^, ^29^ ^]^ According to this relation, upon increasing the diameter of the microsphere, the FSR value gradually decreases with the increase in the number of resonance lines (Figure 6f) and FSR can reach as high as 23 nm for a ≈7.1 µm microresonator. The conventional MFs often suffer from photodegradation when exposed to high‐energy irradiation, leading to a decline in their optical performance.^[^ ^15b^ ^]^ The RCPM matrix plays a crucial role as a protective scaffold for the in situ formation of PAHs. Under continuous laser irradiation of moderate power (50 µW for 532 nm, 2 mW for 785 nm), RCPM‐2 demonstrates consistent WGM emission across the visible to NIR spectrum. Remarkably, even with prolonged exposure of laser excitation, it retains ≈> 92% of its initial PL intensity (Figure S19, Supporting Information). Moreover, the micro‐resonator structures of RCPM remain stable in atmospheric conditions and exhibit resistance to degradation or alteration when subjected to organic solvents or acid treatments, all while maintaining WGM emission (Figure S20, Supporting Information). This protective capability of RCPM, whereby the PAHs are encapsulated within the matrix, serves to shield them from air oxidation through photochemical reactions. Our work presents a pioneering example of utilizing simple, metal‐free PAH assemblies as the basis for an optical microresonator, which exhibits wavelength‐tunable WGMs emission covering the entire DR and NIR spectral regions.

**Figure 7:**
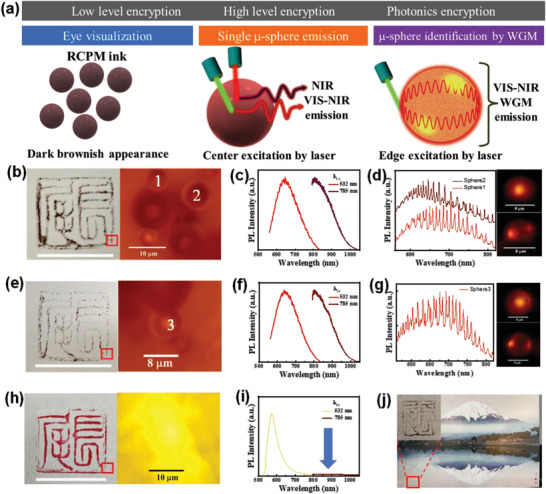
a) Schematic illustration of the hierarchical scheme for anti‐counterfeit application of RCPM‐based ink. The representation includes a digital photograph and microscopic optical imaging, excitation wavelength‐dependent PL spectra, and WGM emissions from an individual microsphere. b,e,h) Digital and magnified optical images of two hanko stamps created using RCPMs‐2, and a control hanko stamp made with dye‐based ink (Scale bar for the hanko stamp is 18 mm). c,f,i) Averaged µ‐PL spectra from ensembles of RCPM‐2 microresonators and dye‐inked paper at 532 and 785 nm laser excitation. d,g) WGM emission from a single microsphere. µ‐PL image achieved by excitation from the center and edge, respectively. j) Application of the RCPM‐2‐based hanko on a commercial painting featuring a panoramic view of Mount Fuji. The inset provides a magnified image of the stamp.

We demonstrated a practical application of RCPM micro‐emitters for encrypted anti‐counterfeit authentication/identification. Counterfeiting remains a significant global challenge, resulting in trillion‐dollar economic losses across various sectors, including contract documents, artistic works, medicine, electronics, and currency.^[^ ^30^ ^]^ Such counterfeiting poses direct risks to economic activity, social life, and security.^[^ ^31^ ^]^ A promising solution lies in highly protective anti‐counterfeiting authentication methods, such as optically integrated verifiable identification (**Figure** **7**a). Unclonable anti‐counterfeit technology, utilizing VIS‐NIR μ‐PL and micro‐resonator‐based WGM emissions, has been successfully demonstrated in the form of hanko name stamps, widely used in East and Southern Asia. In this study, we demonstrated three hanko stamps: two created using the RCPM‐2 ink synthesized from the same batch, and another using a conventional commercial red dye ink. The hanko stamp formed with RCPM‐2 ink shows a distinctive dark brownish hue (Figure 7b,e), contrasting with the red appearance of the dye‐based ink stamp (Figure 7h). Detailed microscopic optical images show the random distribution of μ‐particles in the RCPM ink (Figure 7b,e), introducing a formidable challenge for replication when compared to more conventional dye‐based ink. Owing to excitation wavelength‐dependent PL, microspheres in the RCPM‐2 ink emit both DR‐NIR and NIR light at 532 and 785 nm excitation laser, presenting a dual emission characteristic (Figure 7c,f). This stands in contrast to the dye‐based ink, which emits a single peak in the VIS (Figure 7i). Notably, the specially averaged PL of the two microsphere stamps using RCPM‐2 ink exhibit similarity (Figure 7c,f), offering an intriguing opportunity for additional spectroscopic encryption based on their unique fluorescent properties. Furthermore, by examining *individual* microspheres, spatially resolved µ‐PL reveals unique micro‐resonator behaviors, as evidenced by entirely different resonance peak and Q factor values in μ‐PL spectra (Figure 7g). Laser excitation at 532 nm demonstrates DR‐NIR emission from microsphere centers, while edge excitation shows confined and resonating light on their perimeters due to WGMs. Photonic properties of individual microspheres distinguish two different stamps made of RCPM‐based ink (Figure 7d,g). As a proof of concept, the identity of a Mount Fuji panoramic landscape painting can be assured using the RCPM‐2 ink‐based hanko stamp, effectively differentiating it from other paintings employing this encrypted anti‐counterfeit strategy (Figure 7j). Both the hanko‐stamps on the painting and white paper demonstrate stability in atmospheric conditions and WGM DR‐NIR emission for over six months (Figure S21, Supporting Information). This hierarchically protected authentication provides a robust level of security, capitalizing on the cost‐effectiveness and eco‐friendliness of CPMs synthesized from natural ingredients and a low‐temperature, energy‐saving reaction route.

## 3 Conclusion

In summary, our innovative microresonator, harnessing technology for luminescent carbonized polymer microspheres (CPMs), marks a significant advance in solid‐state fluorescence (SSF) in the DR‐NIR range. Controlled production of red‐emitting CPMs (RCPMs) demonstrates their effectiveness in generating stable solid‐state light emission, providing effective solutions for AIQ concerns. Elongated π‐conjugated PAHs lead to a red shift in light absorption and prolonged NIR emission. Additionally, RCPM spherical shape imparts inherent photonic properties, enabling excitation wavelength‐dependent PL with WGMs up to 1000 nm. This versatile microresonator holds great potential for anti‐counterfeiting labels, seamlessly blending photonics and chemistry for diverse applications.

## 4 Experimental Section

### Materials

NC Corporation (Tokyo, Japan) graciously provided ε‐polylysine (EPL) in a 25 w/v % aqueous solution, with a 36‐mer structure and a molecular weight of ≈4600 g mol^−1^. Citric acid monohydrate (CA) and p‐phenylenediamine (PPD) were procured from FUJIFILM Wako Pure Chemical Corporation, while other reagents were obtained from Wako Pure Chemical Industries (Osaka, Japan) and used without additional refinement.

### Preparation of CPM

In synthesis of YCPMs and RCPMs, a hydrothermal method was employed. For Y‐CPMs, a solution containing 0.15 m of CA and 0.009 m of EPL in 25 mL Milli‐Q water was sonicated for ≈5 min to achieve a transparent, homogeneous solution. The resulting solution was then transferred to a clean autoclave for the hydrothermal reaction. Post‐reaction, solid YCPMs precipitated at the bottom of the Teflon container. Similarly, RCPMs1‐3 were synthesized by adding varying amounts of PPD (0.04, 0.11, and 0.18 m) to a solution containing 0.15 m CA and 0.009 M EPL. The mixture was sonicated for 5 min to obtain a homogeneous solution, followed by the hydrothermal reaction. After the reaction, a dark solid R‐CPM precipitated at the bottom of the Teflon container (Figure S1, Supporting Information). Both YCPMs and RCPMs were subjected to multiple washes with water and ethanol, and then dried in a vacuum furnace at 60 °C for 2 h. The reproducibility of YCPM and RCPM formation is notable, characterized by their precipitation as yellow and dark‐colored materials respectively from the solution (Figure S1b,c, Supporting Information). Details regarding the hydrothermal synthesis process, reaction parameters, and the corresponding sample names are provided in Table S1 (Supporting Information). For comparative purposes, conventional carbon microspheres (CSs) were synthesized hydrothermally from a mixture of CA and sucrose solution through a hydrothermal reaction.

### Preparation of R‐CDs

A solvothermal synthesis was employed to produce R‐CDs at 180 °C for 6 h, utilizing 0.04 m of PPD and a 20 mL solution of N, N‐dimethylformamide. The resulting R‐CDs were subjected to filtration through a 0.2 µm filter to eliminate larger insoluble particles. Subsequently, purification was carried out in an ethanol solution using an osmosis membrane (molecular cutoff = 0.5–1 kDa) for 24 h and replaced the solvent every 4 h interval.

### Characterization

A variety of techniques were utilized to thoroughly examine the microstructure and properties of the materials under investigation. Scanning electron microscopy (SEM) was conducted using a Hitachi model SU‐8000 FE‐SEM and TM3000 table‐top SEM‐EDX, operating at 5–10 kV with Si as the substrate and Pt for coating. Transmission electron microscope (TEM) images were captured using an FEI‐Tecnai G2 operating at 200 kV on carbon‐coated Cu TEM grids. Crystal properties were determined through X‐ray diffraction (XRD) with Cu Kα radiation on a RINT Ultima III instrument from Rigaku Corporation. Composition and chemical bonding states were analyzed using CHNO elemental analysis, X‐ray photoelectron spectroscopy (XPS), solid‐state nuclear magnetic resonance spectroscopy (NMR), and attenuated total reflection‐Fourier transform infrared spectroscopy (ATR‐FTIR). XPS utilized a PHI Quantera SXM with an Al Kα X‐ray source, while ATR‐FTIR measurements were conducted on a Nicolet iS50 FTIR instrument. Solid‐state NMR measurements of ^13^C and ^15^N via cross‐polarization, magic‐angle spinning nuclear magnetic resonance (CPMAS‐NMR) were performed using JEOL/ECA 800 MHz for 13C and JEOL/ECA 500 MHz for ^15^N. During the ^15^N CPMAS analysis, specimens were inserted into a 4 mm PENCIL rotor and rotated at a speed of 10 kHz. Chemical shifts in the resulting ^15^N ssNMR spectra were calibrated externally using CH_3_NO_2_ as a reference at 0 ppm. UV–vis–NIR spectra were recorded with a JASCO (V‐770) spectrometer in reflectance mode. Fluorescence and excitation spectra were measured with a JASCO FP8500DS spectrometer, and absolute quantum yields were determined using a Hamamatsu C9920‐02G integrating sphere system.

### Time Resolved Photoluminescence

Fluorescence decay profiles were recorded using a NanoLog time‐correlated single photon counting (TCSPC) lifetime spectroscopy system from Horiba Jovin Ybon, Japan. The system was equipped with pulsed laser diodes emitting at 361 nm, featuring an average pulse duration of 1.0 ns or less and a frequency of 1 MHz, and 449 nm, with an average pulse duration of 1.2 ns and a frequency of 1 MHz. These laser diodes served as the excitation light source for these experimental procedures.

### Single‐Microsphere μ‐PL Measurements

To study single microspheres emission and WGMs emission, a WITec µ‐PL (WITec alpha300 confocal microscope) system with a Princeton Instruments model Action SP2300 monochromator and an Andor iDus model DU‐401A BR‐DD‐352 CCD camera was used. The YCPM and RCPM samples were dispersed in an alcohol solution for 5 min using sonication. Subsequently, the solution containing microspheres was drop‐cast onto a soda lime glass substrate (SLG) and dried at 50 °C for the examination of the photoluminescence (PL) of individual microspheres. The 50x objective (NA = 0.8, MPLFLN50x, OLYMPUS) was employed in the optical microscope to identify individual microspheres and measure their diameters (d). Various laser sources, including 355 and 470 nm diode pulsed lasers, were employed for photoexcitation. A SuperK Extreme, a pulsed laser, was also used wavelength selectors connected via collimated fiber optic delivery. The wavelengths covered (VIS/NIR) were 400–800/800‐1100, utilizing Acoustic Optic tunable filter technology. The software used for control and programming was SuperKontrol 2.0. This supercontinuum laser source facilitated variable excitation with lasers at 532, 580, and 630 nm for single microspheres emission study. Additionally, a 532 nm Nd:YAG laser source was employed for μ‐PL mapping and spectra of a single microsphere on a SLG substrate. For NIR WGM emission, 785 nm high power single‐frequency diode laser was used from XTRA system, Toptica Photonics AG.

### Computational Methods

In the context of small molecule modeling, calculations for excited states are performed utilizing Time‐Dependent Density Functional Theory (TD‐DFT) reference methods. Various functionals, such as B3LYP and basis sets, like 6–31+G(d), are employed in this framework. To determine absorption properties, molecular structures are initially optimized in the ground state using the relevant functional. Subsequently, vertical excitation energies are computed employing both standard linear‐response and adiabatic approximations.

### Numerical simulation

To simulate the resonance spectra and mode profiles of RCPMs2, a commercial finite‐difference time‐domain (FDTD) solver (Ansys Lumerical) was used. Emission characteristics of RCPMs2 were modeled as a dipole source positioned in the vicinity of the edge of the sphere. Optical power emitted by the dipole was monitored using a transmission box enclosing the dipole. Perfectly matched layers in the x and y directions served as absorbing boundary conditions. RCPMs2 were placed in air (n = 1, k = 0), and optical constants of these spheres were set to n = 1.457 and k = 0.003, giving a good agreement with experimental spectra.

### Unclonable Whispering‐Gallery Anti‐Counterfeit Emission

In formulating anti‐counterfeit ink, 50 mg of RCPMs‐2 were combined with 0.9 mL of H_2_O and 0.1 mL of a 3 wt.% polyvinyl alcohol (PVA) solution. The resulting ink was then applied to a hanko stamp and stamped onto white paper. The measurement of WGM PL in the DR to NIR wavelength region of individual microspheres took place under 532 nm Nd:YAG laser excitation by using a long distance 50x objective (LD EC Epiplan‐Neofluar 50x/0.55, ZEISS) in optical microscope. Furthermore, the ink was tested on a corner of an A3‐sized Mount Fuji portrait (Made in Japan, Interior Wallpaper) that was purchased from Amazon.co.jp.

## Conflict of Interest

The authors declare no conflict of interest.

## Supporting information

Supporting Information

## Data Availability

The data that support the findings of this study are available in the supplementary material of this article.
