# Supplementary material for: Rare‐Earth‐Metal‐Free Solid‐State Fluorescent Carbonized‐Polymer Microspheres for Unclonable Anti‐Counterfeit Whispering‐Gallery Emissions from Red to Near‐Infrared Wavelengths
Source: Adv Sci (Weinh). 2024 Jun 12;11(30):2400693. doi: 10.1002/advs.202400693 (PMC11321640; doi:10.1002/advs.202400693)
Supplement: Supplementary file 1 — Supporting Information [file ADVS-11-2400693-s001.pdf]

ADVANCED SCIENCE
Open Access

Supporting Information

for *Adv. Sci.*, DOI 10.1002/advs.202400693

Rare-Earth-Metal-Free Solid-State Fluorescent Carbonized-Polymer Microspheres for Unclonable Anti-Counterfeit Whispering-Gallery Emissions from Red to Near-Infrared Wavelengths

Barun Kumar Barman, Hiroyuki Yamada, Keisuke Watanabe, Kenzo Deguchi, Shinobu Ohki, Kenjiro Hashi, Atsushi Goto and Tadaaki Nagao**

Supporting Information

Rare-Earth-Metal-Free Solid-State Fluorescent Carbonized-Polymer Microspheres for Unclonable Anti-Counterfeit Whispering-Gallery Emissions from Red to Near-Infrared Wavelengths

Barun Kumar Barman [1*], Hiroyuki Yamada [1], Keisuke Watanabe [1], Kenzo Deguchi [2], Shinobu Ohki [2], Kenjiro Hashi [3], Atsushi Goto [3], and Tadaaki Nagao [1, 4*]

[1] Research Center for Materials Nanoarchitectonics (WPI-MANA), National Institute for Materials Science (NIMS), Tsukuba, Ibaraki 305-0044, Japan

[2] Research Network and Facility Services Division, National Institute for Materials Science (NIMS), 3-13 Sakura, Tsukuba, Ibaraki 305-0003, Japan

[3] Center for Basic Research on Materials, National Institute for Materials Science (NIMS), 3-13 Sakura, Tsukuba, Ibaraki 305-0003, Japan

[4] Department of Condensed Matter Physics Graduate School of Science, Hokkaido University, Sapporo 060-0810, Japan

Corresponding authors E-mail: barman.kumarbarun@nims.go.jp, NAGAO.Tadaaki@nims.go.jp

Table S1. Synthesis of different types of CPM via hydrothermal synthesis

Sample name	Experimental conditions					Morphology
	CA (M)	EPL (M)	PPD (M)	Temperature (^oC)	Time (h)	
YCPM	0.15	0.009	0	180	4	Microsphere
RCPM-1	0.15	0.009	0.04	180	4	Microsphere
RCPM -2	0.15	0.009	0.11	180	4	Microsphere
RCPM -3	0.15	0.009	0.18	180	4	Microsphere
R-CDs	0	0	0.04	180	6	Colloidal solution

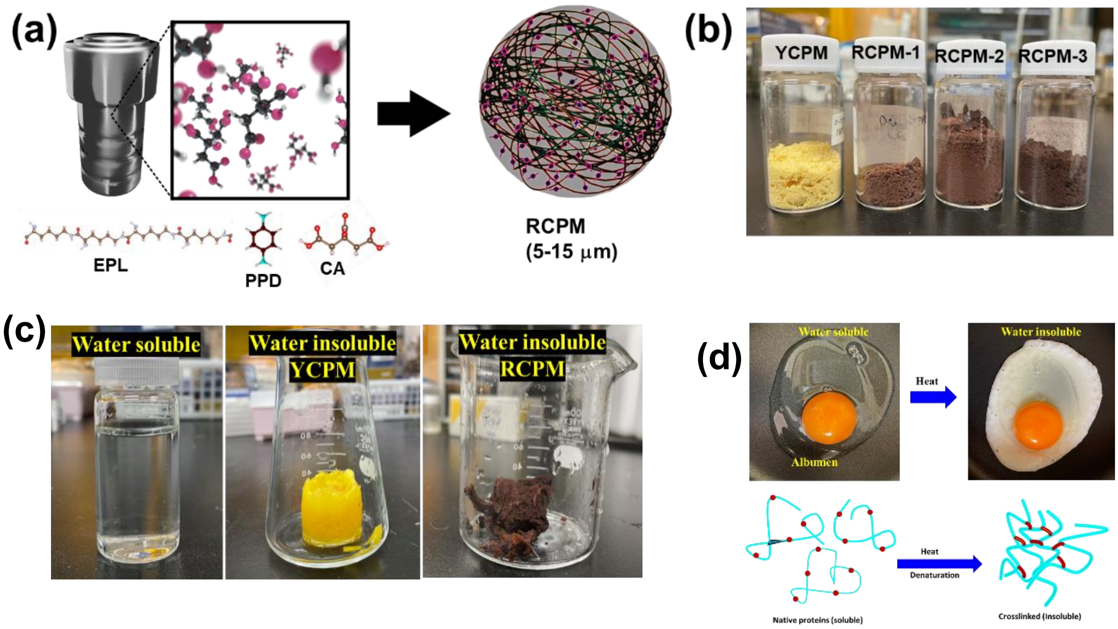

Figure S1. (a and b) Illustration of the gram-scale synthesis of CPM using the hydrothermal method, accompanied by corresponding digital photographs. (c) shows the formation of water insoluble YCPM and RCPM from their soluble raw ingredients. (d) shows the digital photographs and schematic representation of protein denaturation in albumen (egg white) due to heat treatment, followed by cross-linking of unfolded proteins, leading to changes in structure and properties.

Figure S2

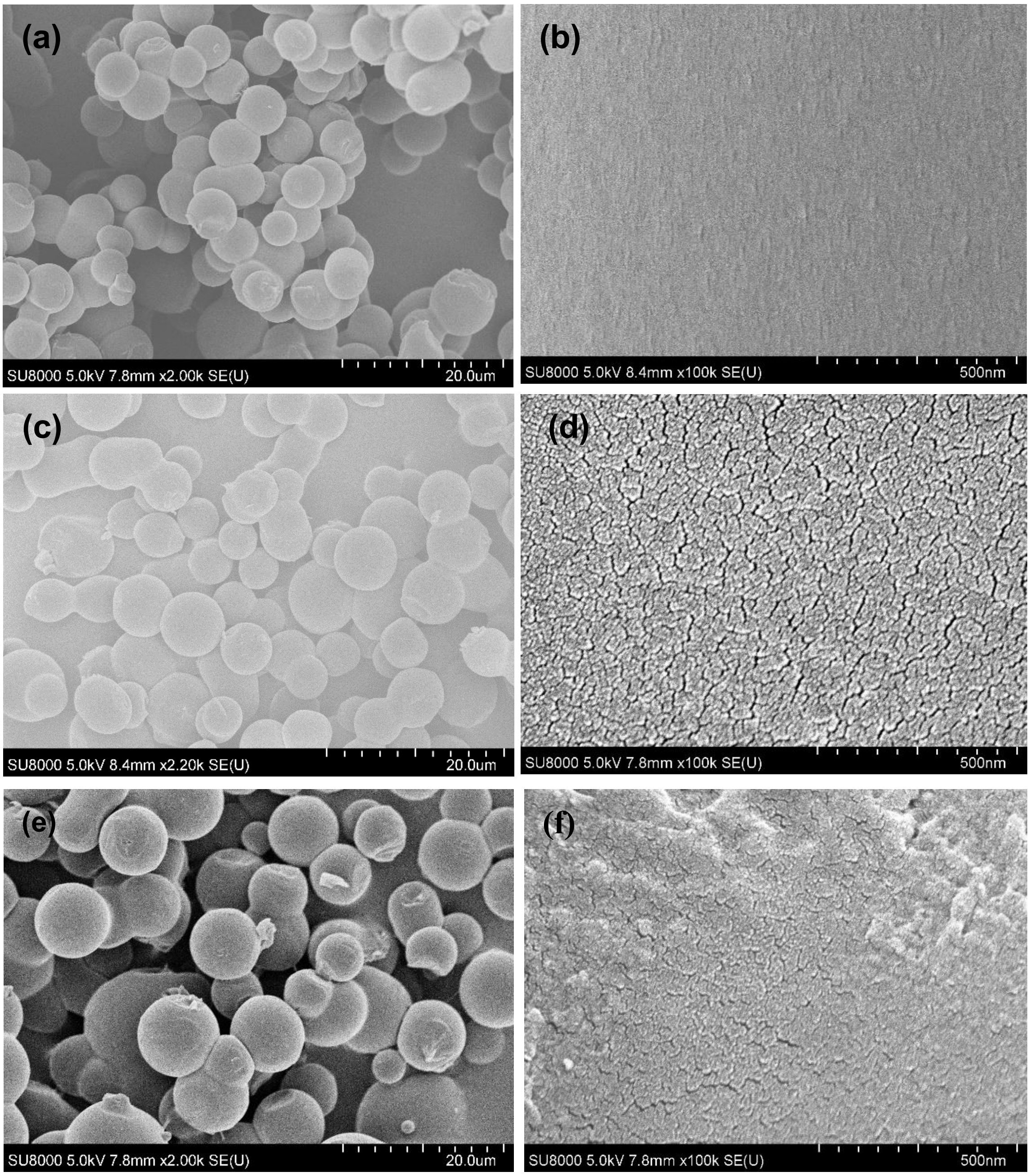

Figure S2. SEM images showing the surface morphology of YCPM (a, b), RCPM-1 (c, d), and RCPM-3 (e, f) at both low and high magnifications. The microstructures showcase the assembly of CDs nanoparticles.

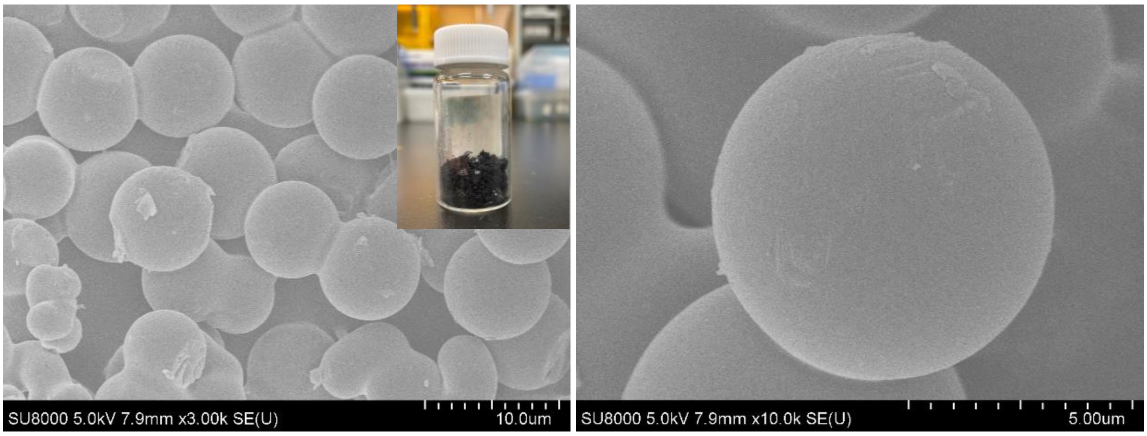

Figure S3. (a and b) SEM images show the surface morphology of carbon sphere (CS) derived by hydrothermal treatment of CA (0.12 M) + sucrose (0.45 M) solution for 6 h (Inset photograph shows the black-colored CS).

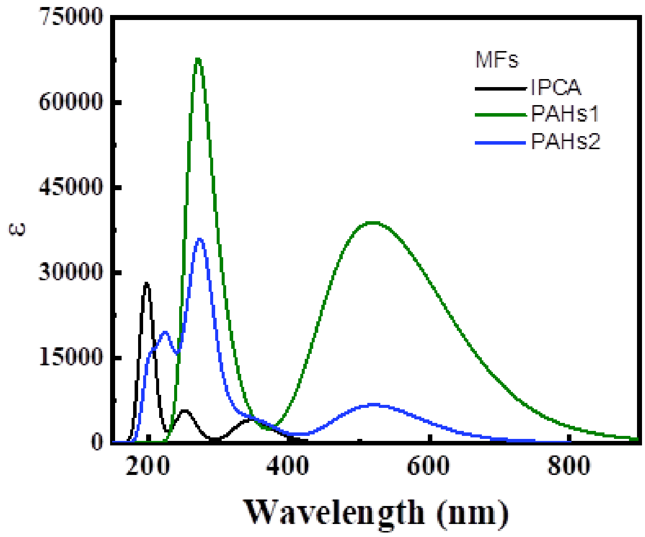

Figure S4. shows the simulated absorbance spectra of small molecular fluorophores of IPCA derivatives and various types of red-emitting PAHs. The YCPM contains the IPCA MF derivative corresponding to high UV absorbance, while the gradual inclusion of PAHs into the YCPM leads to a red shift in absorbance due to broad absorbance in the VIS to NIR to spectral region for RCPM.

(a)

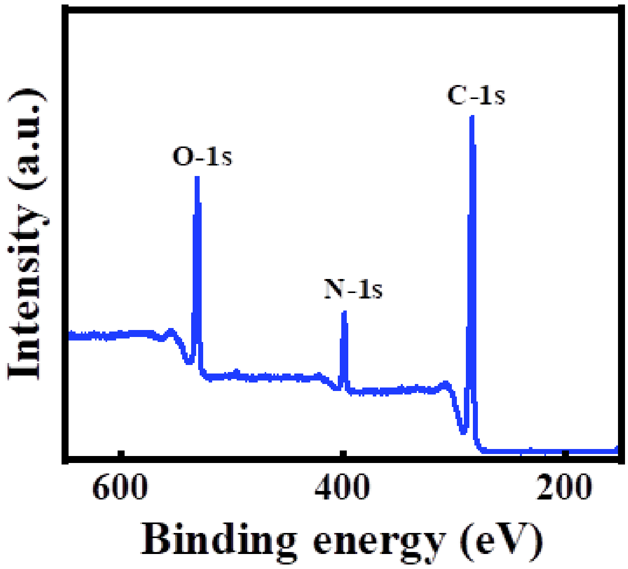

(b)

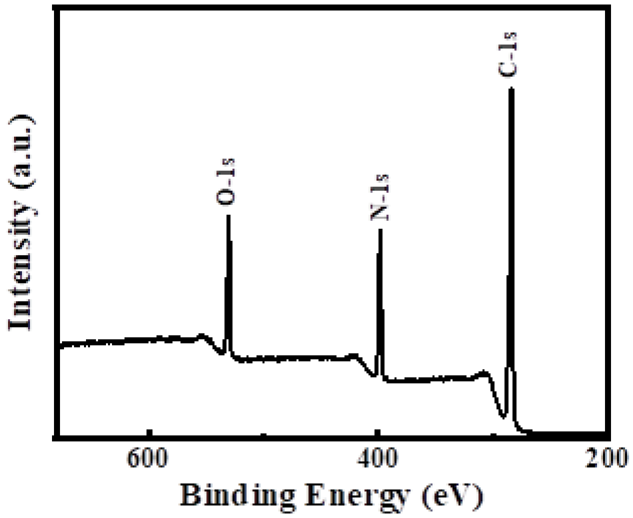

Figure S5. (a and b) XPS survey spectra of YCPM and ε-polylysine (EPL) linear peptides.

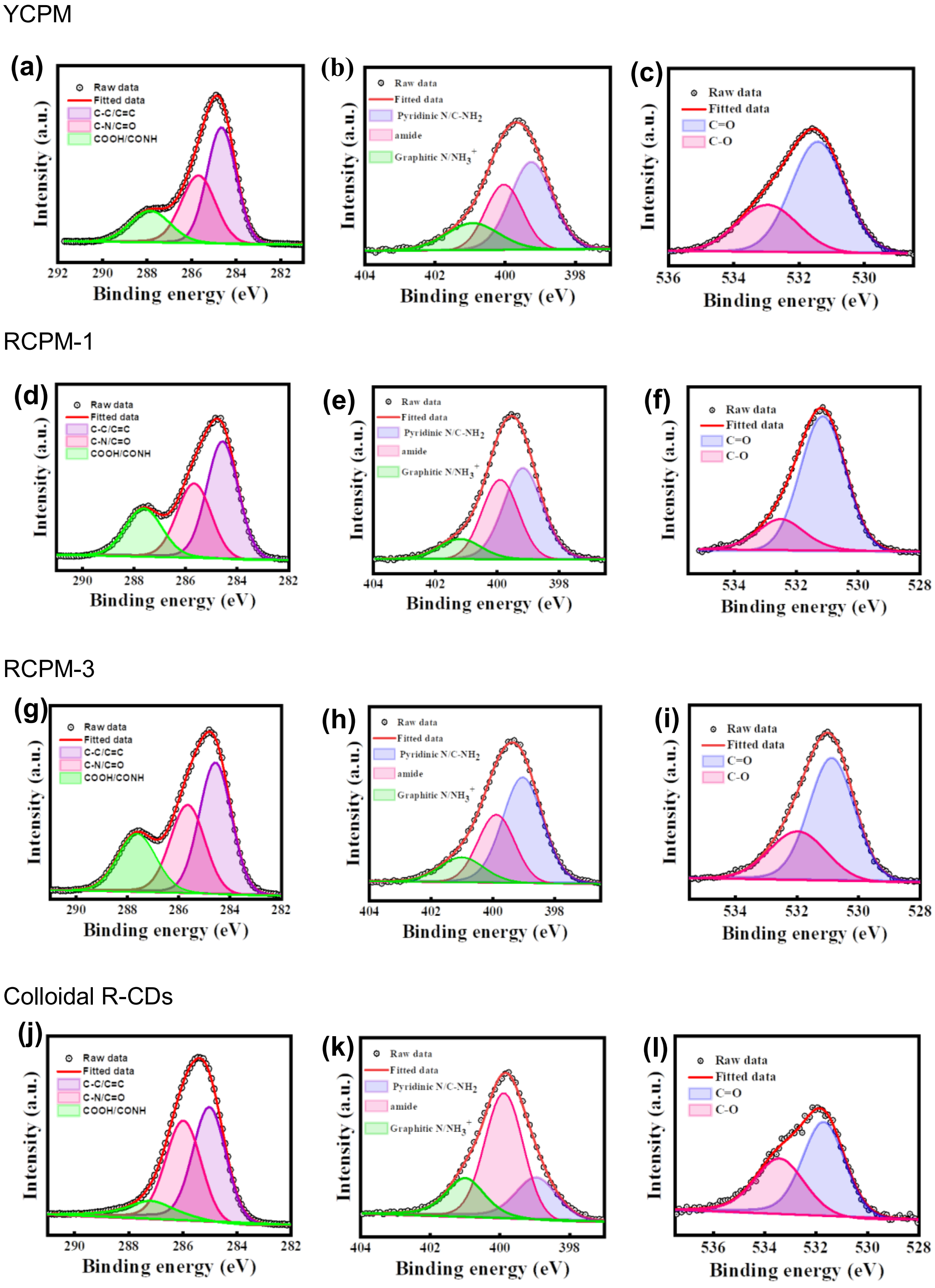

Figure S6. The HRXPS spectra have been deconvoluted to investigate the different functional groups of C-1s, N-1s, and O-1s in YCPM (a-c), RCPM-1 (d-f), RCPM-3 (g-i), and R-CDs (j-l). This analysis provides insights into the various functionalization and elemental composition of these micro to nanostructures.

Table S2: Comparative composition of C, O, and N and N/C ratio of different samples.

Samples name	C	N	O	N/C
EPL	72	16.2	11	0.225
YCPM	70.3	11.7	18	0.17
RCPM-1	69.26	15.38	15.36	0.22
RCPM-2	68.69	16.04	15.27	0.23
RCPM-3	68.75	16.31	14.93	0.24
R-CDs	82.88	11.29	5.26	0.14

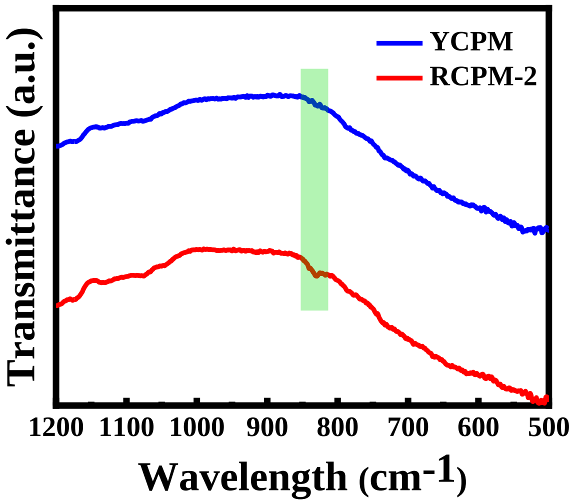

Figure S7. Shows the magnified ATR-FTIR spectra of YCPM and RCPM-2. The appearance of a small new peak at 825 cm^{-1} may indicate the formation of PAH ring structures in RCPM during the incorporation of PPD into the reaction medium.

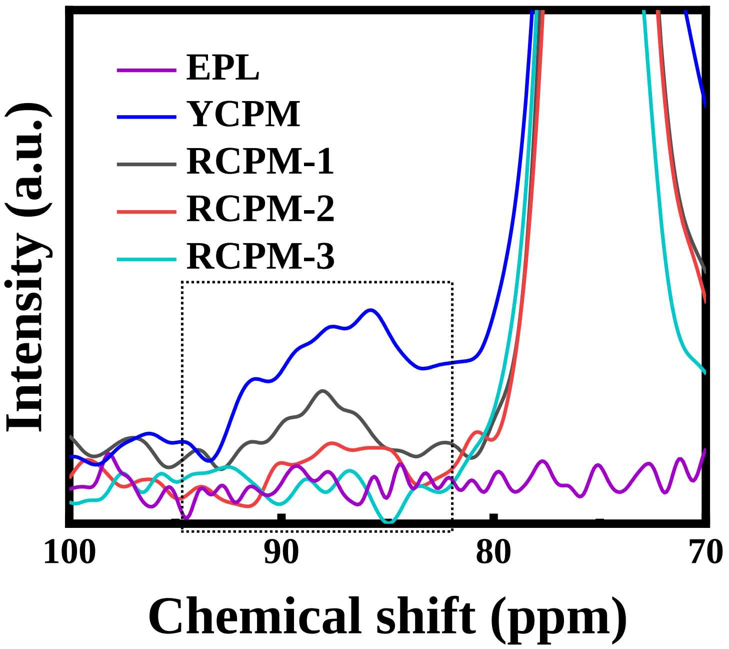

Figure S8. Shows the magnified solid-state ^{13}C CPMAS NMR spectra of different samples and in the range of 100 to 70 ppm chemical shift.

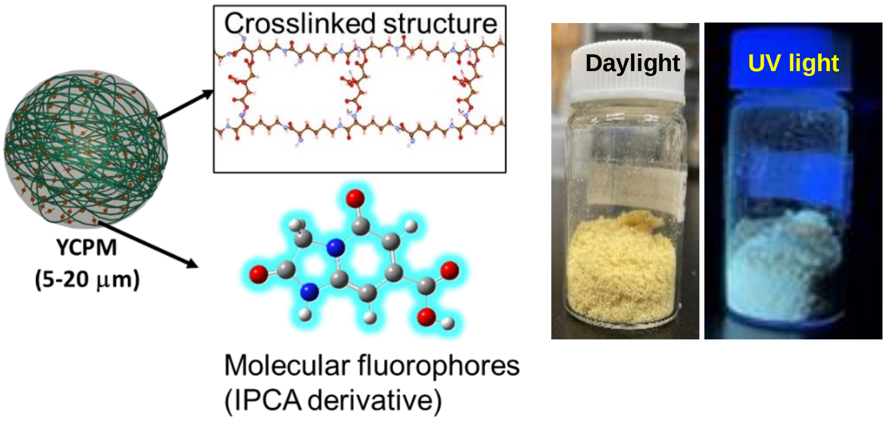

Figure S9. A schematic representation illustrating the formation of YCPM without the addition of PPD. This process results in the formation of microstructures that integrated with the in-situ formed IPCA derivative (molecular fluorophores). Subsequently, the YCPM exhibit a predominant absorption of UV to blue wavelength and emit blue light by UV excitation.

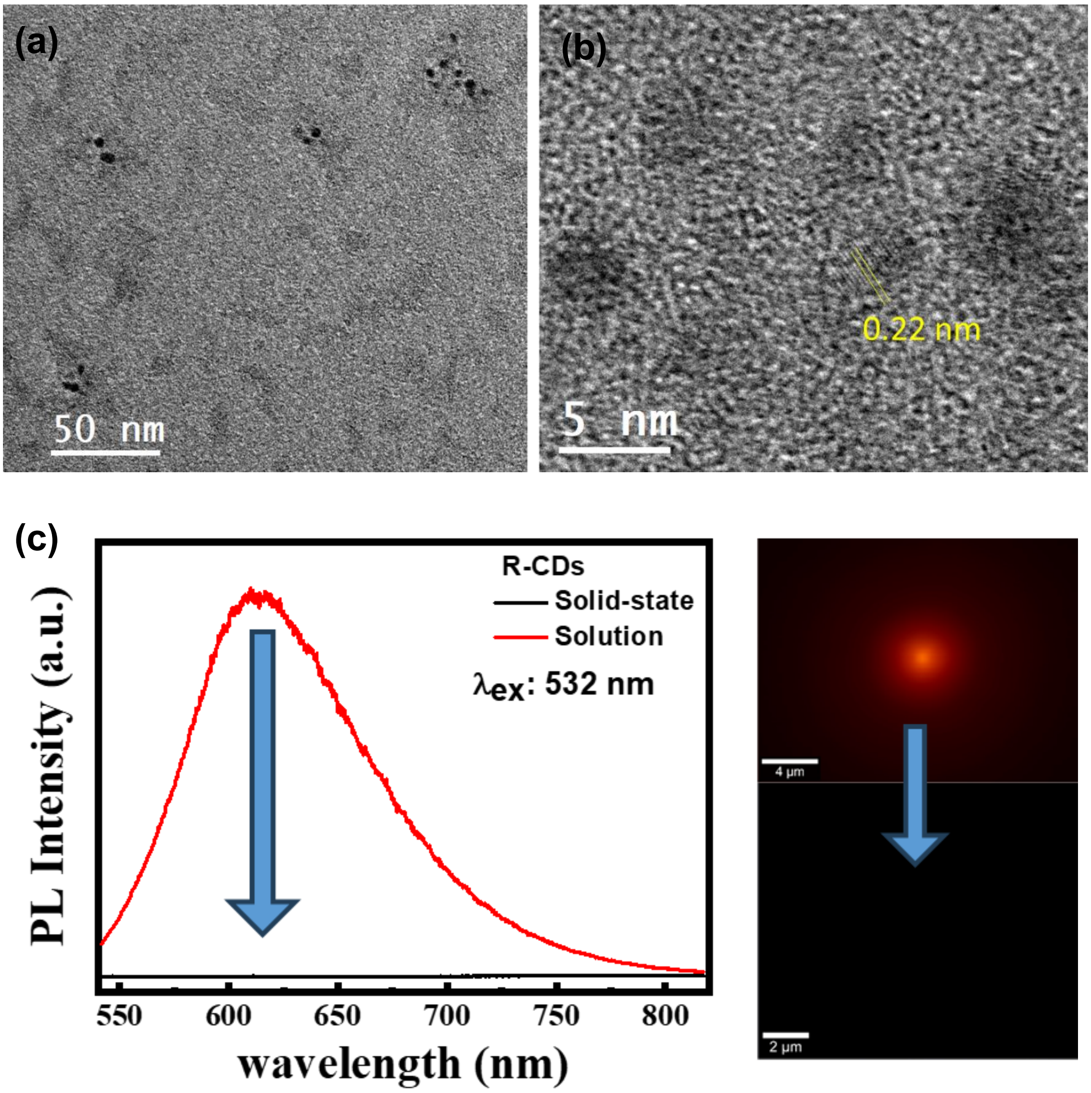

Figure S10. (a and b) TEM and THRTEM images of R-CDs colloids synthesized through solvothermal methods using PPD as precursors, along with their colloidal and solid-state photoluminescent (PL) behaviors and corresponding micro-PL (μ-PL) images. TEM images validate the generation of small crystalline R-CDs during the solvothermal reaction of PPD. The μ-PL spectrum and images affirm that R-CDs exhibit red emission in colloidal form but undergo complete quenching of their emission due to aggregation in the solid state.

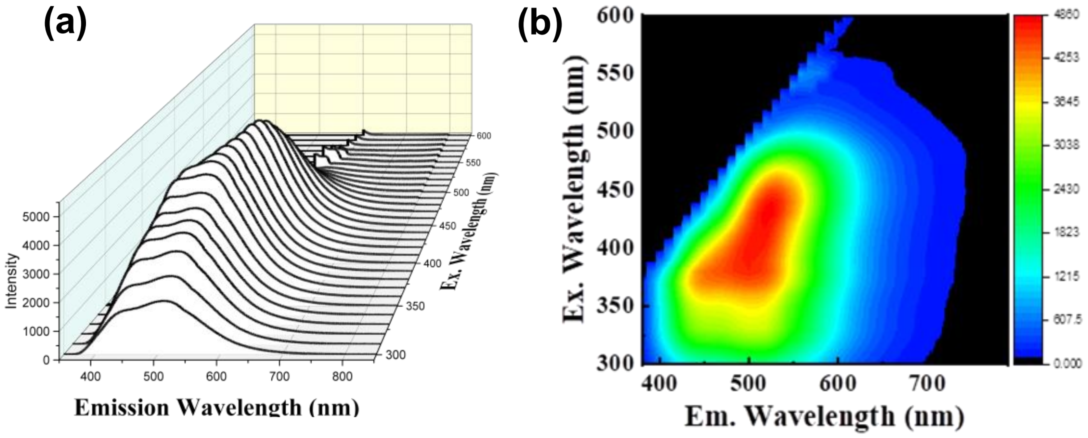

Figure S11. (a and b) display the 3-D PL spectrum and excitation-emission matrix (EEM) of YCPM. Both spectra indicate strong cyan emission under UV excitation and exhibit multicolor emission ranging from cyan to red when varying the excitation wavelength. However, emissions at higher wavelengths are weaker compared to cyan and green emissions.

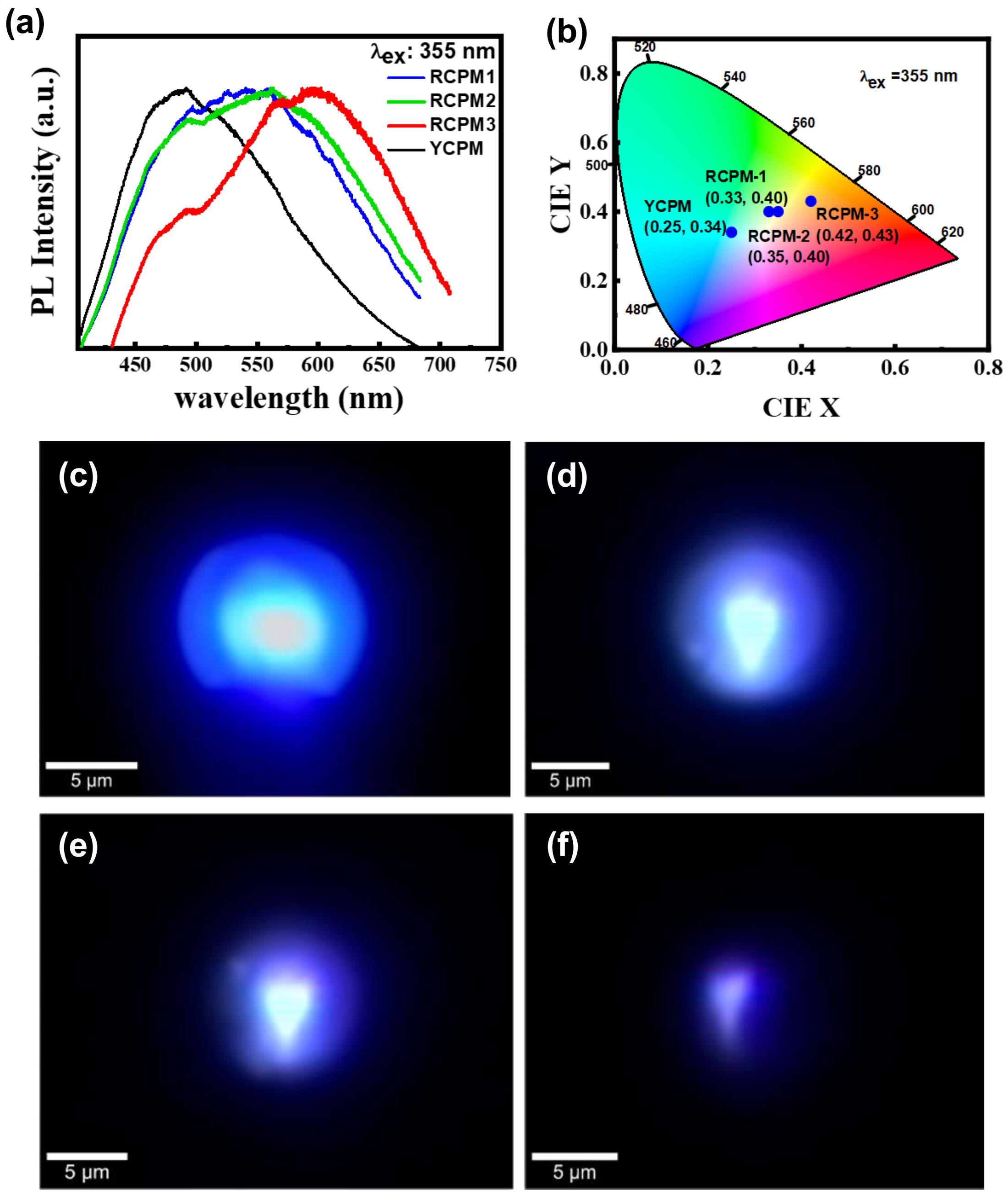

Figure 12. (a and b) show the μ-PL spectrum, along with the corresponding CIE coordinates, of YCPM and RCPM1-3 is presented under 355 nm laser excitation at the microsphere center. (c-f) shows corresponding μ-PL images. The spectrum, coordinates, and images collectively demonstrate the progression from cyan to white light emission, highlighting the gradual enhancement of red-light emitting PAHs.

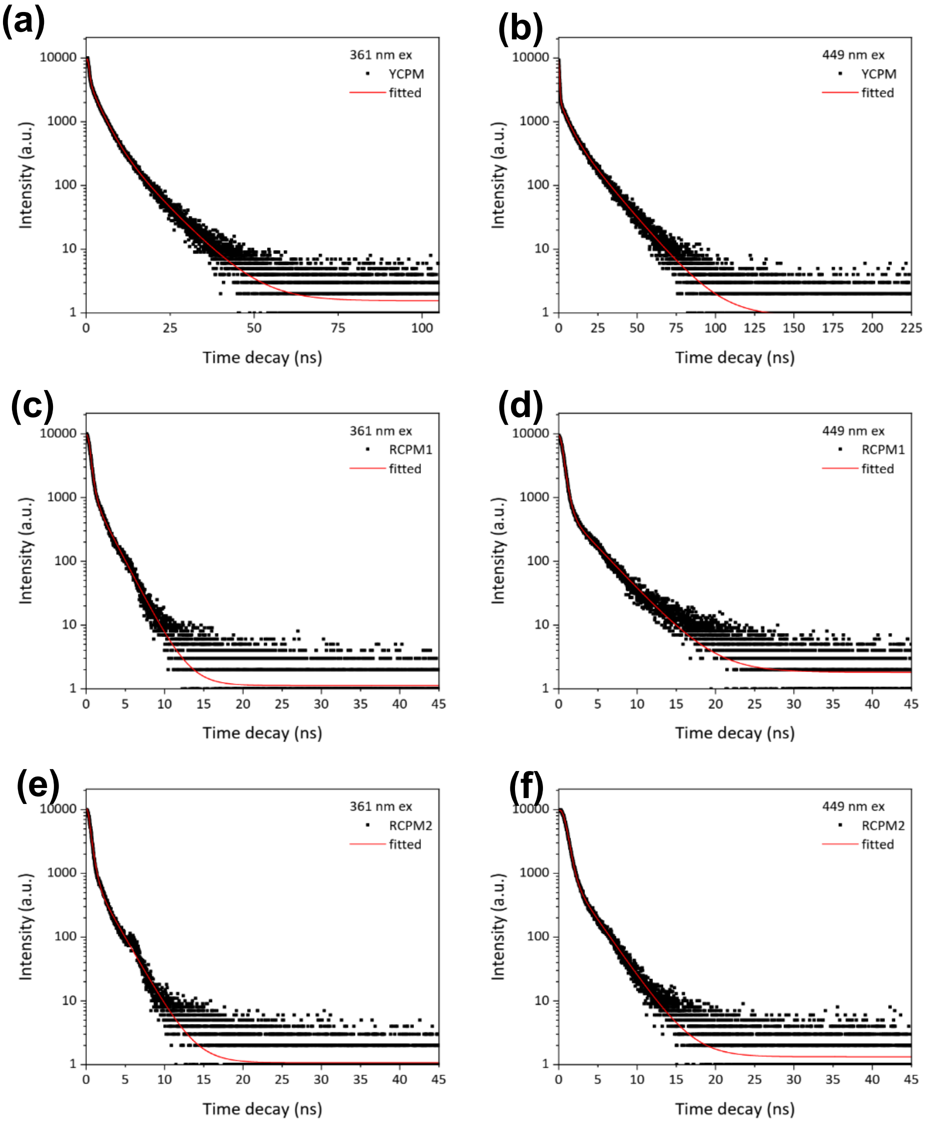

Figure 13. The time-resolved photoluminescence (TRPL) spectra were acquired for YCPM and RCPM-1, RCPM-2 by laser excitations at 361 nm and 449 nm. This comprehensive analysis offers valuable insights into the emission dynamics and characteristics of these materials, particularly in the context of different excitation wavelengths and the underlying Förster Resonance Energy Transfer (FRET) mechanism.

Time-resolved photoluminescence (TRPL) and average lifetimes (τ_{avg}): The measured decay spectra were fitted with the bi/tri-exponential functions, which are expressed by the equation 1 and the $_{avg}$ express by the following equations 2, respectively.[1]

$$I(t) \approx A_1 exp\left(-\frac{t}{\tau_1}\right) + A_2 exp\left(-\frac{t}{\tau_2}\right) + A_3 exp\left(-\frac{t}{\tau_3}\right) \ldots\ldots\ldots\ldots (1)$$

where τ_1, τ_2, and τ_3 are the first, second, and third components of the PL lifetime, and A_1, A_2, and A_3 are the amplitudes of each component, respectively.

$$\tau_{avg} = \sum_{i=1}^{n} A_i \tau_i \ldots\ldots\ldots\ldots\ldots\ldots\ldots\ldots\ldots\ldots\ldots\ldots\ldots\ldots\ldots (2)$$

Table S3: TRPL and photophysical parameters of different CPM including excitation wavelength and average lifetimes (τ_{avg}).

Sample	Excitation wavelength (nm)	PL peak (nm)	τ_1 (ns)	A_1	τ_2 (ns)	A_2	τ_3 (ns)	A_3	τ_{avg} (ns)
YCPM	361	448	3.10	0.467	8.08	0.2614	0.39	0.2716	3.665736
	449	535	5.14	0.2416	15.0	0.3983	0.36	0.3601	7.34596
RCPM-1	361	433	0.32	0.7452	1.83	0.2548			0.704748
	449	577	0.42	0.8288	3.32	0.1712			0.91648
RCPM-2	361	433	0.39	0.7853	2.02	0.2147			0.739961
	449	582	0.46	0.8077	2.47	0.1923			0.846523

FRET Mechanism study: FRET is a process that entails the transfer of energy, and this transfer is inversely related to the sixth power of the distance (r) between the donor and acceptor molecules. The efficiency of FRET (EFRET) is mathematically expressed as by $E_{FERT} = \frac{1}{1+\left(\frac{r}{R_\circ}\right)^6}$ where $R_\circ$ represents the characteristic distance for the donor-acceptor pair, marking the point at which EFRET reaches a value of 0.5.[2] Experimental techniques for detection include quenching of donor fluorescence and sensitized acceptor fluorescence. In the realm of steady-state measurements, FRET efficiencies (E) are determined by the formula $E = 1 - \frac{\tau_{DA}}{\tau_D}$, where τ_{DA} and τ_D denote the donor fluorescence lifetimes in the presence and absence of the acceptor, respectively.[3] This approach provides insights into the intricate dynamics of energy transfer at the molecular level.

(a)

(b)

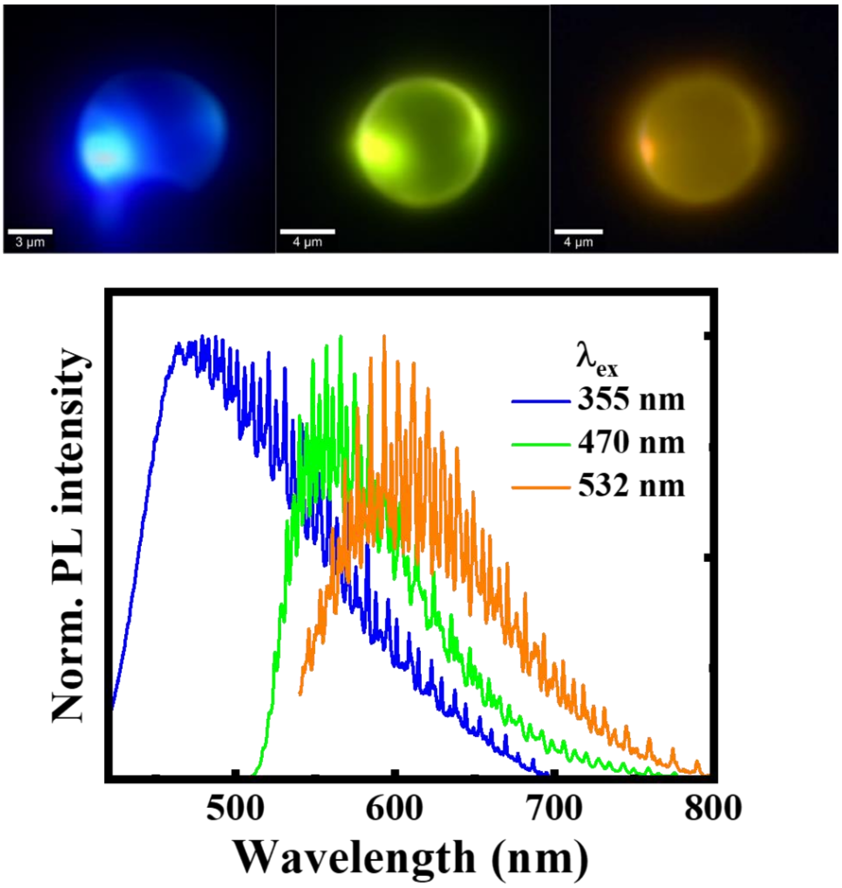

Figure S14. (a and b) Show the μ-PL image and corresponding spectrum demonstrating WGM emissions from an individual YCPM microsphere across the cyan to red wavelength range by varying the excitation laser source.

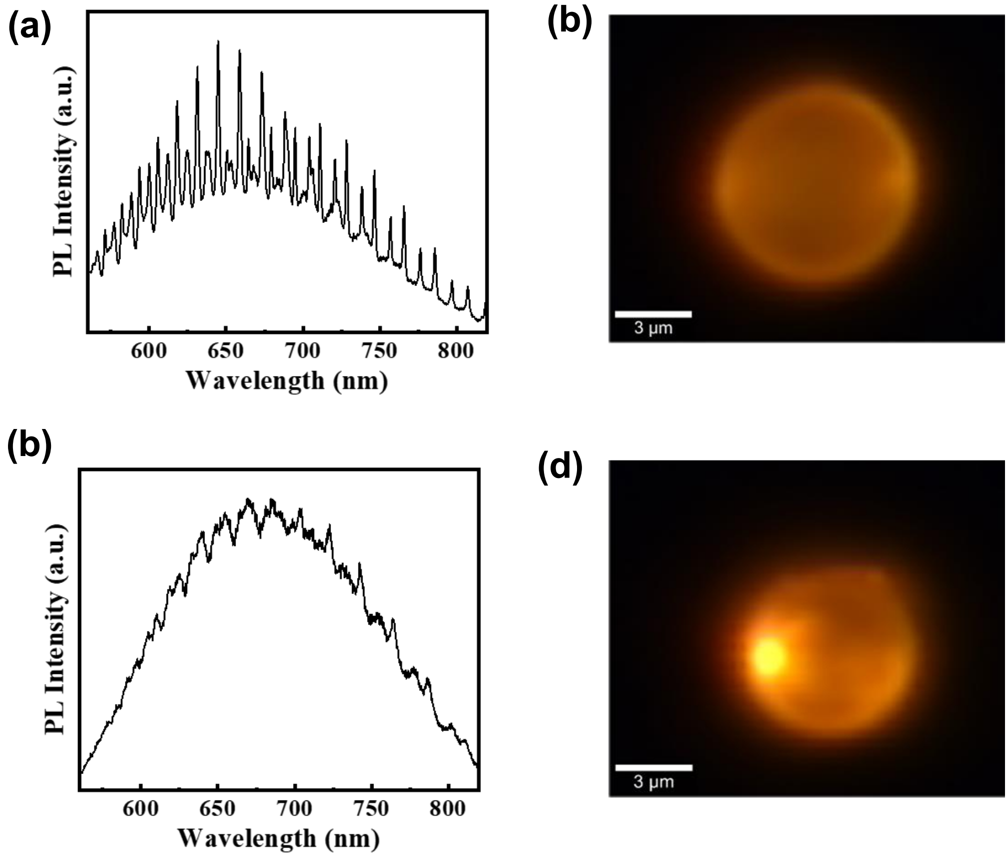

Figure S15. WGMs emission spectra from a single microsphere and corresponding μ-pl images from RCPM-1 (a, b) and RCPM-3 (c, d) under 532 nm laser excitation at their edges.

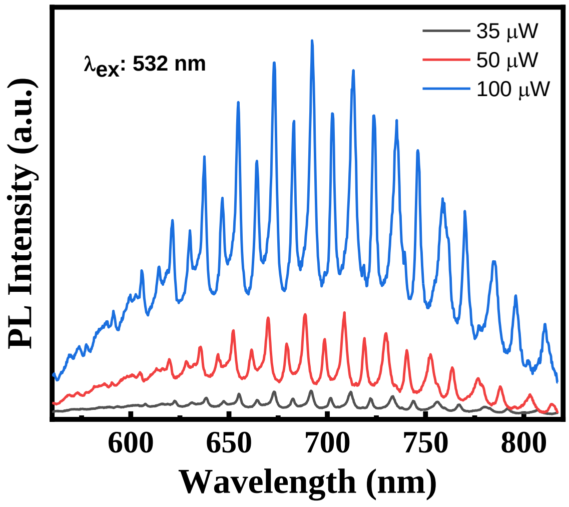

Figure S16. Laser power dependent WGM emission spectra of a single microsphere from RCPM-2.

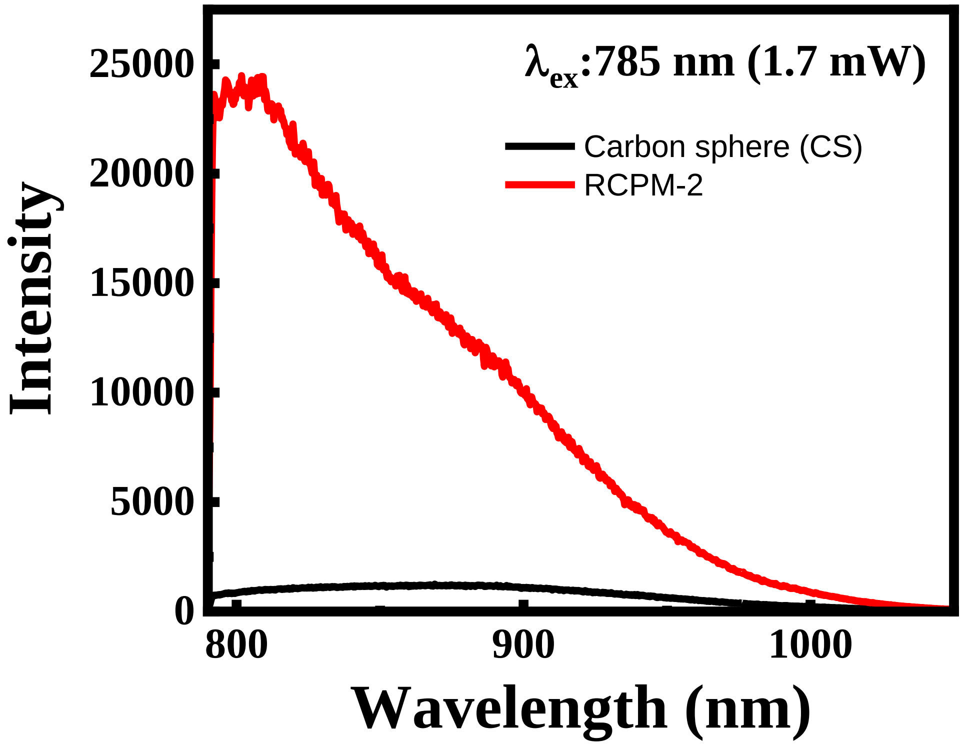

Figure S17. illustrates the photoluminescence (PL) spectrum for individual carbon microspheres (CS) obtained via hydrothermal treatment of a sucrose+ CA solution. The spectrum is contrasted with that of RCPM-2, and measurements were performed with a 1.7 mW 785 nm laser power. The comparative PL spectrum reveals that typical CS exhibit relatively weak near-infrared (NIR) emission, whereas RCPM-2 demonstrates nearly 20 times higher NIR emission.

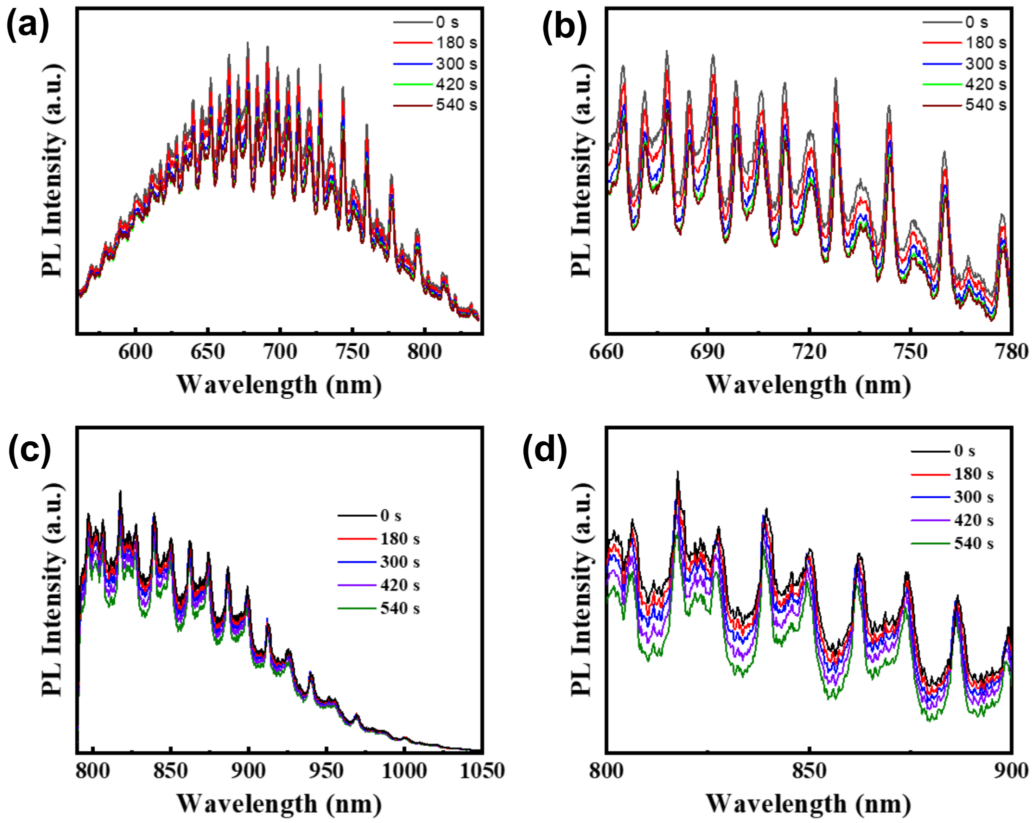

Figure S18. WGM emission and corresponding magnified spectra for prolonged continuous 532 nm laser (a and b) and 785 nm laser (c and d) excitation of RCPM-2.

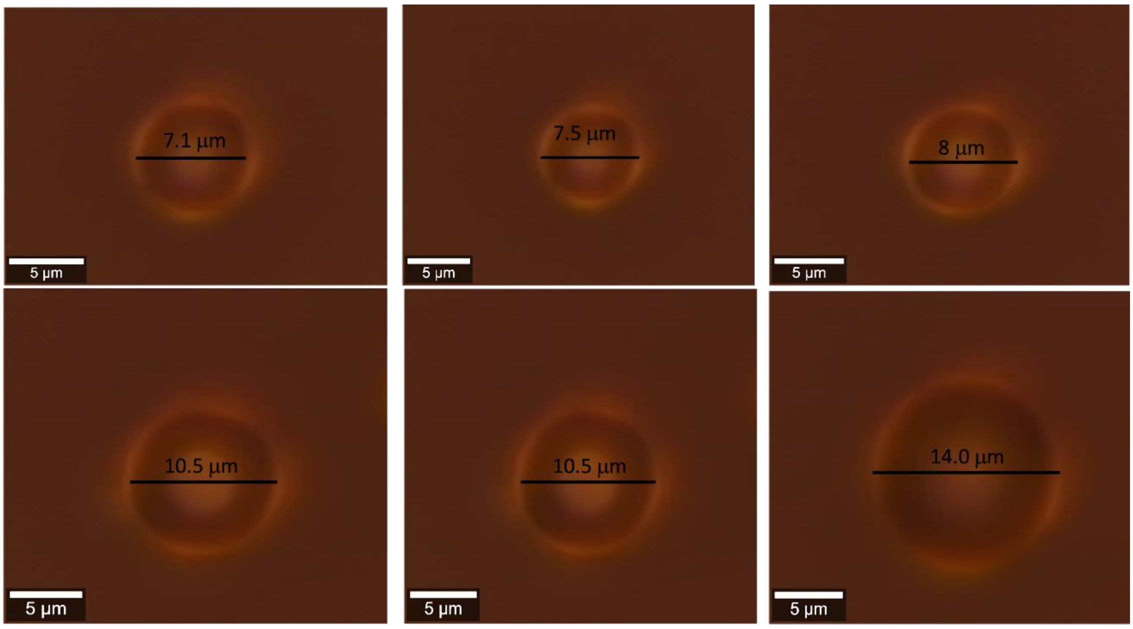

Figure S19. displays optical images of RCPM-2 utilized for the emission of NIR WGMs, along with the corresponding diameter determination of Free Spectral Range (FSR).

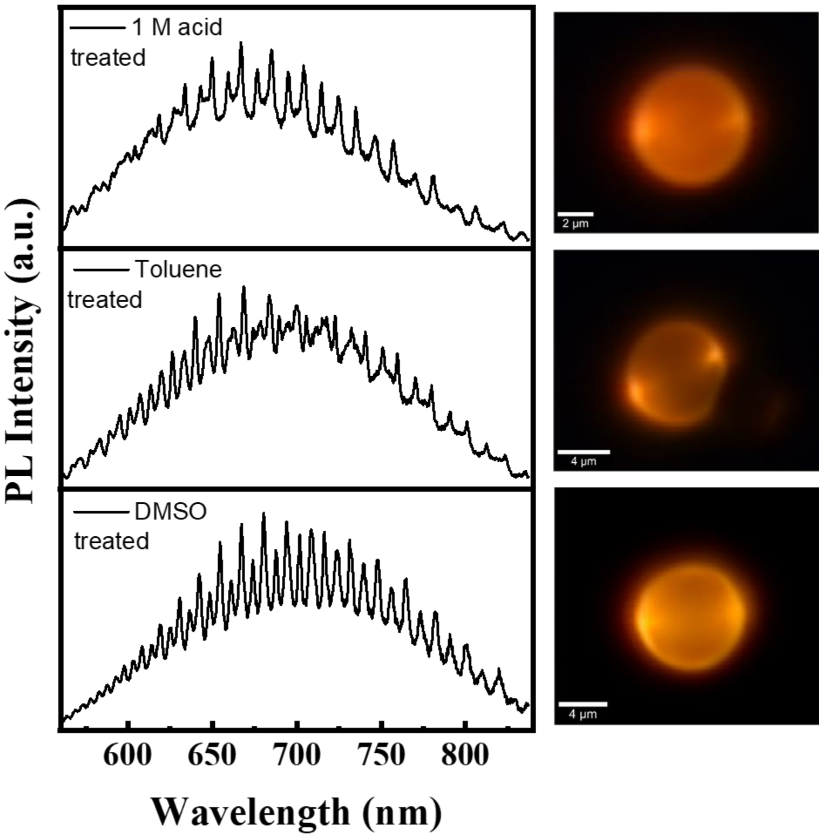

Figure S20. The WGMs emission spectra of a single microsphere from RCPM-2 were observed under different conditions, including treatment with organic solvent and HCl acid. These conditions were varied to assess their impacts on the WGM emission spectra.

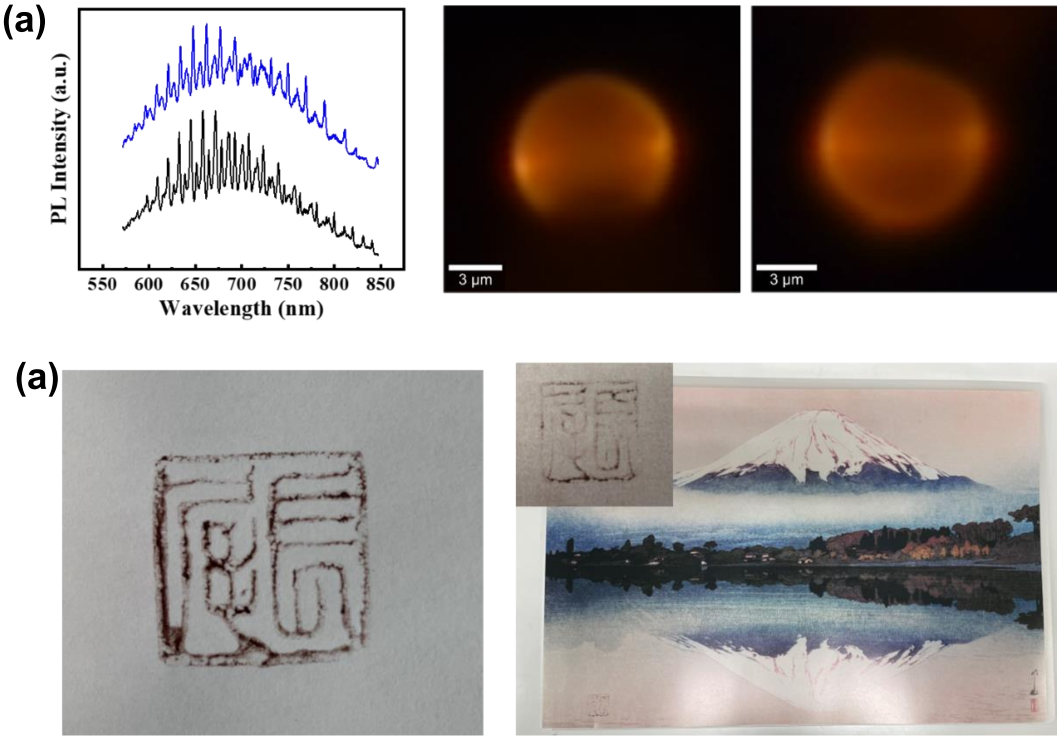

Figure S21. (a) μ-PL WGM emission from two individual microspheres under 532 nm laser excitation from a hanko stamp on paper and corresponding μ-PL images after approximately 6 months. (b) Digital photographs of the hanko stamp on paper and Mount Fuji portrait after approximately 6 months. This study indicates the stability of the hanko stamp made with RCPM-2-based ink.

Table S4: Overview of carbon nanodots (CNDs) and their aqueous ink formulations for anti-counterfeit applications, contrasted with the security strategy of RCPM for authentication.

**Materials**	**Synthesis**	**Counterfeit strategy**	**PL responsive**	**Security level**	**Ref**
NCDs	Hydrothermal (CA+EDA)	Excitation dependent PL	λ_{ex} = 365 nm, Blue PL	Low	[4]
CDs	Pyrolysis of turtle shells	colloidal photonic crystals (CPCs)/CDs based structural color andfluorescenc	λ_{ex} = 365 nm, Blue PL and angular dependence of structural colors	High	[5]
NCDs	Hydrothermal (CA+EDA)	FL lifetime-encoded anti-counterfeiting	Blue FL (λ_{ex} = 365 nm) and yellow in FL lifetime imaging mode	High	[6]
$NaYF_4$:Er,Yb (Tm)/CDs	Hydrothermal	Up-conversion (UC) and dc-conversion (DC) fluorescent	Dual-mode fluorescence patterns Blue FL (λ_{ex} = 365 nm) Green FL (λ_{ex} = 980 nm)	High	[7]
NCDs	Sonochemical (Gelatine)	Tunable emission	Invisible in daylight, yellow (λ_{ex} = 532 nm) and red FL (λ_{ex} = 633 nm) on laser irradiation	Medium	[8]
CDs	CA+urea heating	Tunable emission	Blue and green and red emission from different CDs	Medium	[9]
CNDs	Microwave (CA+urea)	Excitation wavelength dependent PL	Blue to yellow λ_{ex} = 340 to 500 nm Colourless to green PL λ_{ex} = blue light colourless to red FL (λ_{ex} = green light)	Medium	[10]
N, S co-doped CQDs	Lignin and 2,4-diaminobenzene sulfonic acid derived	anti-counterfeiting ink	The yellowish green-emissive	Low	[11]

CNDs	Solid-state reaction (Urea and diammonium hydrogen citrate)	Excitation wavelength dependent FL	Colourless to Green FL (λ_{ex} = 365 nm)	Low	[12]
NCNDs	Hydrothermal (CA and tris(hydroxymethyl) methyl aminomethane (Tris))	Secret information communication	Colourless to Blue FL (λ_{ex} = 365 nm)	Low	[13]
CDs	Hydrothermal (Mushrooms and 5-amino-2-methylphenol)	Excitation-independent PL and anti-counterfeiting ink	Green emission at 519 nm (λ_{ex} =467)	Low	[14]
CNDs	Hydrothermal (Trimellitic acid)	Advanced anti-counterfeiting and information encryptiondecryption with water-stimulir responsive yellow RTP	Blue FL (λ_{ex} = 365 nm) and yellow RTP	High	[15]
CNDs	Solvothermal (Citric acid and urea)	Bright multicolour CL	Blue, green and red CL in dark	High	[16]
CDs fiber	Polystyrene heating followed by drawing by metal strips	random lashing	Blue lasing	High	[17]
S, N co-doped CNDs	Hydrothermal (CA and cysteine)	FL ink	Bright blue FL (λ_{ex} = 365 nm)	Low	[18]
CDs Phosphor-Al_2O_3 Hybrids	Solvothermal treatment	Room temperature phosphorescence (RTP)	Blue fluorescence and green RTP emissions Yellow RTP under irradiation with white light	High	[19]
GQDs in layered double hydroxides	EDTA pyrolysis	Dual-emission characteristics	FL and RTP (that is, dual-mode emission)	High	[20]

S, N co-doped CNDs	Solvothermal (Melamine and DTSA)	Two-switch mode luminescence with dual encryption	Blue FL (λ_{ex} = 365 nm) pink (λ_{ex} =365 nm and red FL (λ_{ex} =254 nm) on drying after adding water	High	[21]
CNDs	Solvothermal (m-Phenylenediamines)	Triple-mode (PL, UCPL and RTP)	Bright blue PL (λex. = 365 nm), blue-green RTP and cyan UCPL (λex = 800 nm)	High	[22]
GQDs	Solvothermal of Graphite Intercalation Compounds	Room-temperature phosphorescence (RTP) and thermally activated delayed fluorescence (TADF)	Blue PL and green RTP/TADF	High	[23]
C, P co-doped CNDs	Polymerization (Glucose and L-aspartic acid)	Matrix-free RTP which disappears on water spray	Blue FL (λ_{ex}= 365 nm) with green afterglow	High	[24]
CNDs	Pyrolysis (Ethylenediaminetetraacetic acid disodium salt)	RTP	White FL (λ_{ex} = 365 nm) and green RTP	Medium	[25]
N-CNDs	MW (Citric acid and ammonia)	RTP, Thermal treatment controlled multilevel. FL/phosphorescence	Blue FL (λ_{ex} = 365 nm) and green RTP after UV off	Medium	[26]
N, P codpped CNDs	Solvothermal (Diethylenetriamine pentaacetic Acid)	Visible light-excited URTP, not-easily replicable, double-encryption mode	Blue FL (λ_{ex} = 365 nm) and yellow green URTP	High	[27]
NCD	Hydrothermal (cellulose and urea)	Invisible FL inks	Blue FL (λ_{ex} = 365 nm)	Low	[28]
N, P codpped CNDs	Microwave (Ethanolamine and phosphoric Acid)	URTP for advanced anti-counterfeiting and information protection	Blue FL (λ_{ex} = 365 nm) and a green URTP under UV off	High	[29]
RCPM	Hydrothermal (Citric acid and poly-lysine	1. Random distribution of RCPM	Deep red to NIR fluorescence and WGM	High and impossible	This work

	+ PPD)	2. The multiple emission bands from VIS-NIR<br>4. Whispering Gallery Mode (WGM) emission in VIS-NIR emission, with each microsphere exhibiting distinct microresonance patterns. This layer-by-layer protection renders replication impossible, ensuring unclonable anti-counterfeiting and information protection.	emission,<br>(λex. = 532 and 785 nm)		

CDs: Carbon dots, CQD: carbon quantum dots, CNDs: carbon Nano dots, NCDs: N doped carbon dots, CPD: carbonized polymer dots, GQDs: Graphene quantum dots, CA: Citric acid monohydrate, PPD: p-Phenylenediamine, EDAT: Ethylenediaminetetraacetic acid, CL: Cathodoluminescence, DTSA: dithiosalicylic acid.

References:

[1] a) K. Nemoto, J. Watanabe, H.-T. Sun, N. Shirahata, *Nanoscale* **2022**, *14* (27), 9900, https://doi.org/10.1039/D2NR02071H; b) Y. Altıntas, M. Y. Talpur, M. Ünlü, E. Mutlugün, *The Journal of Physical Chemistry C* **2016**, *120* (14), 7885, https://doi.org/10.1021/acs.jpcc.6b01977.

[2] P. Liao, S. Zang, T. Wu, H. Jin, W. Wang, J. Huang, B. Z. Tang, Y. Yan, *Nature Communications* **2021**, *12* (1), 5496, https://doi.org/10.1038/s41467-021-25789-9.

[3] D. S. Biswas, P. Gaki, E. Cruz Da Silva, A. Combes, A. Reisch, P. Didier, A. S. Klymchenko, *Advanced Materials* **2023**, *35* (29), 2301402, https://doi.org/https://doi.org/10.1002/adma.202301402.

[4] B. Kumar Barman, T. Nagao, K. K. Nanda, *Applied Surface Science* **2020**, *510*, 145405, https://doi.org/https://doi.org/10.1016/j.apsusc.2020.145405.

[5] J. Guo, H. Li, L. Ling, G. Li, R. Cheng, X. Lu, A.-Q. Xie, Q. Li, C.-F. Wang, S. Chen, *ACS Sustainable Chemistry & Engineering* **2020**, *8* (3), 1566, https://doi.org/10.1021/acssuschemeng.9b06267.

[6] S. Kalytchuk, Y. Wang, K. Poláková, R. Zbořil, *ACS Applied Materials & Interfaces* **2018**, *10* (35), 29902, https://doi.org/10.1021/acsami.8b11663.

[7] M. Li, W. Yao, J. Liu, Q. Tian, L. Liu, J. Ding, Q. Xue, Q. Lu, W. Wu, *Journal of Materials Chemistry C* **2017**, *5* (26), 6512, https://doi.org/10.1039/C7TC01585B.

[8] C. Li, X. Sun, Y. Li, H. Liu, B. Long, D. Xie, J. Chen, K. Wang, *ACS Omega* **2021**, *6* (4), 3232, https://doi.org/10.1021/acsomega.0c05682.

[9] S. Ren, B. Liu, M. Wang, G. Han, H. Zhao, Y. Zhang, *Journal of Materials Chemistry C* **2022**, *10* (31), 11338, https://doi.org/10.1039/D2TC02664C.

[10] S. Qu, X. Wang, Q. Lu, X. Liu, L. Wang, *Angewandte Chemie International Edition* **2012**, *51* (49), 12215, https://doi.org/https://doi.org/10.1002/anie.201206791.

[11] L. Zhu, D. Shen, Q. Wang, K. H. Luo, *ACS Applied Materials & Interfaces* **2021**, *13* (47), 56465, https://doi.org/10.1021/acsami.1c16679.

[12] W. U. Khan, D. Wang, W. Zhang, Z. Tang, X. Ma, X. Ding, S. Du, Y. Wang, *Scientific Reports* **2017**, *7* (1), 14866, https://doi.org/10.1038/s41598-017-15054-9.

[13] Y. Liu, L. Zhou, Y. Li, R. Deng, H. Zhang, *Nanoscale* **2017**, *9* (2), 491, https://doi.org/10.1039/C6NR07123F.

[14] Q. Duan, Y. He, X. Long, J. Wang, C. Ni, S. Wu, *Advanced Sustainable Systems* *n/a* (n/a), 2300536, https://doi.org/https://doi.org/10.1002/adsu.202300536.

[15] K. Jiang, X. Gao, X. Feng, Y. Wang, Z. Li, H. Lin, *Angewandte Chemie International Edition* **2020**, *59* (3), 1263, https://doi.org/https://doi.org/10.1002/anie.201911342.

[16] C.-L. Shen, Q. Lou, C.-F. Lv, J.-H. Zang, S.-N. Qu, L. Dong, C.-X. Shan, *Advanced Science* **2019**, *6* (11), 1802331, https://doi.org/https://doi.org/10.1002/advs.201802331.

[17] Y. Ni, H. Wan, W. Liang, S. Zhang, X. Xu, L. Li, Y. Shao, S. Ruan, W. Zhang, *Nanoscale* **2021**, *13* (40), 16872, https://doi.org/10.1039/D1NR04707H.

[18] Y. Zhang, J. He, *Physical Chemistry Chemical Physics* **2015**, *17* (31), 20154, https://doi.org/10.1039/C5CP03498A.

[19] D. Lu, K. Lu, H.-T. Wen, Z. Wei, A. Bianco, G.-G. Wang, H.-Y. Zhang, *Small* **2023**, *19* (31), 2207046, https://doi.org/https://doi.org/10.1002/smll.202207046.

[20] L. Bai, N. Xue, Y. Zhao, X. Wang, C. Lu, W. Shi, *Nano Research* **2018**, *11* (4), 2034, https://doi.org/10.1007/s12274-017-1820-z.

[21] H. Yang, Y. Liu, Z. Guo, B. Lei, J. Zhuang, X. Zhang, Z. Liu, C. Hu, *Nature Communications* **2019**, *10* (1), 1789, https://doi.org/10.1038/s41467-019-09830-6.

[22] K. Jiang, L. Zhang, J. Lu, C. Xu, C. Cai, H. Lin, *Angewandte Chemie International Edition* **2016**, *55* (25), 7231, https://doi.org/https://doi.org/10.1002/anie.201602445.

[23] M. Park, H. S. Kim, H. Yoon, J. Kim, S. Lee, S. Yoo, S. Jeon, *Advanced Materials* **2020**, *32* (31), 2000936, https://doi.org/https://doi.org/10.1002/adma.202000936.

[24] Y. Gao, H. Han, W. Lu, Y. Jiao, Y. Liu, X. Gong, M. Xian, S. Shuang, C. Dong, *Langmuir* **2018**, *34* (43), 12845, https://doi.org/10.1021/acs.langmuir.8b00939.

[25] Y. Deng, D. Zhao, X. Chen, F. Wang, H. Song, D. Shen, *Chemical Communications* **2013**, *49* (51), 5751, https://doi.org/10.1039/C3CC42600A.

[26] Z. Tian, D. Li, E. V. Ushakova, V. G. Maslov, D. Zhou, P. Jing, D. Shen, S. Qu, A. L. Rogach, *Advanced Science* **2018**, *5* (9), 1800795, https://doi.org/https://doi.org/10.1002/advs.201800795.

[27] Y. Gao, H. Zhang, S. Shuang, C. Dong, *Advanced Optical Materials* **2020**, *8* (7), 1901557, https://doi.org/https://doi.org/10.1002/adom.201901557.

[28] B. V, A. S. Pillai, K. P. Surendran, R. T.P.D., S. K.I., *ChemistrySelect* **2024**, *9* (4), e202303346, https://doi.org/https://doi.org/10.1002/slct.202303346.

[29] K. Jiang, Y. Wang, X. Gao, C. Cai, H. Lin, *Angewandte Chemie International Edition* **2018**, *57* (21), 6216, https://doi.org/https://doi.org/10.1002/anie.201802441.
